# Diving into the Depths: Uncovering Microplastics in Norwegian Coastal Sediment Cores

**DOI:** 10.1021/acs.est.4c04360

**Published:** 2024-09-11

**Authors:** Fangzhu Wu, Karin A. F. Zonneveld, Hendrik Wolschke, Robin von Elm, Sebastian Primpke, Gerard J. M. Versteegh, Gunnar Gerdts

**Affiliations:** † Alfred-Wegener-Institut Helmholtz-Zentrum für Polar- und Meeresforschung, Biologische Anstalt Helgoland, Kurpromenade 201, 27498 Helgoland, Germany; ‡ MARUM - Centre for Marine Environmental Sciences, University of Bremen, 28359 Bremen, Germany; § Department of Geosciences, University of Bremen, 28359 Bremen, Germany; ∥ Environmental Radiochemistry, Institute of Coastal Environmental Chemistry, Helmholtz-Zentrum Hereon, 21502 Geesthacht, Germany; ⊥ Department of Physics and Earth Sciences, Constructor University, 28759 Bremen, Germany

**Keywords:** microplastics, vertical profiles, sediment core, dating, Norwegian Coastal Current

## Abstract

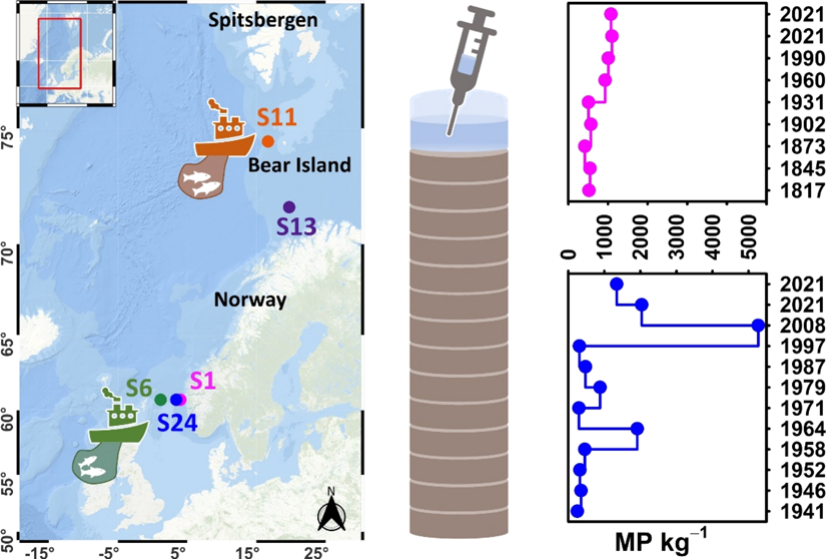

High concentrations of microplastics (MPs) have been documented in the deep-sea surface sediments of the Arctic Ocean. However, studies investigating their high-resolution vertical distribution in sediments from the European waters to the Arctic remain limited. This study examines MPs in five sediment cores from the Norwegian Coastal Current (NCC), encompassing the water-sediment interface and sediment layers up to 19 cm depth. Advanced analytical methods for MP identification down to 11 μm in size were combined with radiometric dating and lithology observations. MPs were present across all sediment cores, including layers predating the introduction of plastics, with concentrations exhibiting significant variation (54–12,491 MP kg^–1^). The smallest size class (11 μm) predominated in most sediment layers (34–100%). A total of 18 different polymer types were identified across all sediment layers, with polymer diversity and depth correlations varying widely between stations. Our findings suggest that differences in seafloor topography and the impact of anthropogenic activities (e.g., fishing) lead to varying environmental conditions at the sampling sites, influencing the vertical distribution of MPs. This challenges the reliability of using environmental parameters to predict MP accumulation zones and questions the use of MPs in sediment cores as indicators of the Anthropocene.

## Abstract 2

Our study reveals the prevalence of microplastics in layers from Norwegian coastal sediment cores, including those from years before plastic mass production, challenging their use as an indicator of the Anthropocene Epoch.

## 1 Introduction

The concept of the “Anthropocene” proposes a geological epoch marked by significant human influence on Earth’s systems.^1^ While not officially recognized as a geological term as of March 2024, it is informally used to describe the era of anthropogenic impact on geological processes.^2^ Various proposals suggest the mid-20th century “Great Acceleration” as a key period, characterized by population growth, industrialization, and intensified mineral and energy use.^3^ Near-synchronous stratigraphic markers, such as artificial radionuclides and aluminum metal, are considered indicative of the onset of this era.^3^ Discussions also explore the potential use of plastics as stratigraphic indicators.^4−9^

Since the first fully synthetic plastic, “Bakelite”, was invented in 1907, it revolutionized the industry with its heat-resistant and electrically insulating properties.^10,11^ This marked the advent of the age of synthetic polymers. The post-World War II (WWII) period witnessed rapid growth in the plastics industry, with annual plastic production increasing from 2 million metric tons (Mt) in 1950^12^ to 400.3 Mt by 2022.^13^ Meanwhile, the emission of plastic waste into the world’s oceans has significantly increased.^14^ Exposure to sunlight, mechanical abrasion, and temperature fluctuations cause plastic items to degrade, breaking into smaller pieces in the environment, such as microplastics (MPs, <5 mm^15^).^16,17^ MPs have penetrated every compartment of the ocean, from the surface to the deepest seabed, and from the poles to the coastlines of the most remote islands.^18−21^ They are detectable in organisms as small as plankton to those as large as whales, posing significant threats to marine ecosystems.^14,22−24^

The ocean bottoms, representing Earth’s most widespread habitat, support high biodiversity and key ecosystem services.^25^ However, the seabed also serves as the largest known reservoir for plastic debris, with high quantities of MPs observed in deep-sea sediments.^19,26−28^ MPs, including positively buoyant polymers, could sink and deposit on the seafloor, influenced by biofouling, adherence of particles, and other biological processes.^29−31^ Significant concentrations of MPs have been documented in the deep-sea surface sediments (top 5 cm) within the Arctic Ocean,^27,28^ with only one study reporting MP distribution in one sediment core up to a depth of 10 cm.^32^

The Norwegian Coastal Current (NCC), part of the larger North Atlantic Current (NAC) system, flows along the western coast of Norway, transporting water, nutrients, and sediments from the North Sea toward the Arctic Ocean.^33,34^ However, studies investigating the stratification of MP deposition in sediments along the NCC from the European waters to the Arctic remain limited. In this study, we present the first assessment of the vertical distribution of MPs in five sediment cores collected in the study area (depth up to 19 cm). Our findings provide a comprehensive overview of MP concentrations, polymer compositions, and size distributions, particularly in conjunction with the dating of radionuclides (^210^Pb, ^137^Cs), an age-depth model, and the lithology of the sediments. This information is essential for predicting the movement of MPs and assessing their long-term environmental persistence. Additionally, our findings may challenge the practice of using MPs as a stratigraphic marker to denote sediment strata of the Anthropocene.

## 2 Materials and Methods

### 2.1 Sediment Core Sampling

**Figure 1:**
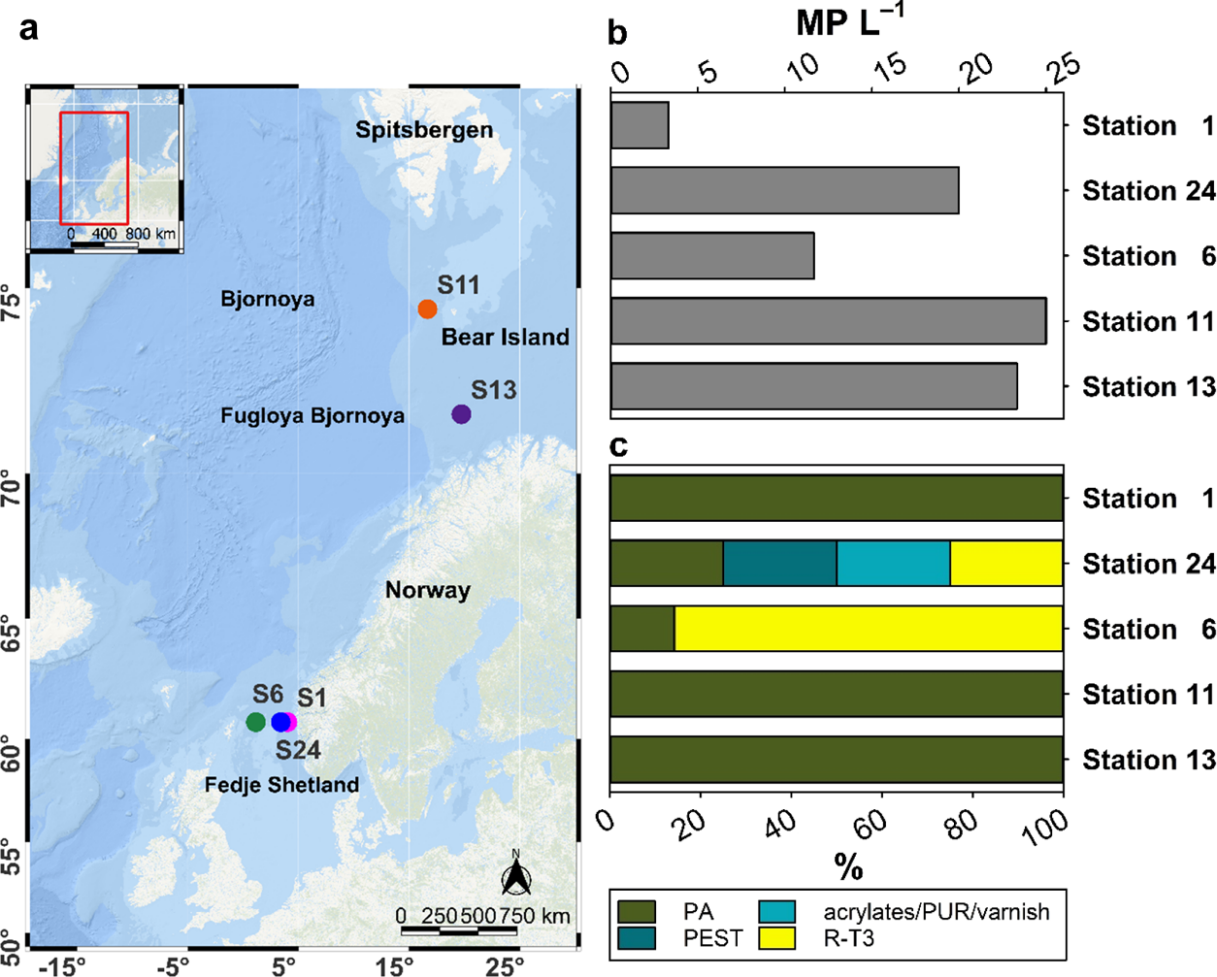
(a) Geographical locations of the sampling stations. (b) Concentrations of microplastic particles (in MP L^–1^) in the overlying water in each core. (c) Polymer composition in the overlying water in each core. polyamide (PA), polyester (PEST), polyurethane (PUR), and rubber type 3 (R-T3).

A total of five sediment samples were collected (water depth, 144–320 m) during the cruise He578 on board the research vessel (RV) *Heincke*(35) (4th June–seventh July 2021). Three samples were collected at the Fedje/Shetland transect (stations S1, S24, and S6). Two were collected within the Arctic Circle (stations S11 and S13), from Bjornoya W (near Bear Island) and the Fugloya Bjornoya transect, respectively (Figure 1a, geographical coordinates details see Table S1, Supporting Information (SI)). To obtain intact samples, a multiple corer (MUC) equipped with a combination of eight poly(vinyl chloride) (PVC) tubes and four stainless steel metal tubes was used (Figure S1). The metal tubes were employed in collecting sediment samples for MP analysis to prevent plastic contamination resulting from potential scratching on the inner surfaces of PVC tubes.^36^ For balance reasons, metal tubes were placed at the four outer positions (Cores 1–4), while PVC tubes were placed in the middle. Metal tubes exhibited superior recovery compared to plastic tubes, as they sealed more effectively and, in contrast to the plastic tubes, demonstrated no sediment loss when retrieving sandy sediments containing pebbles. Following the retrieval of the MUC, 200 mL of the water from the overlying water-sediment interface in Core 1 was carefully collected using a glass syringe (100 mL, Carl Roth GmbH+Co. KG, Germany). The top 3 cm of the sediments from Cores 1–3 were then sliced off with a metal spatula. Core 1 from each station was subsequently sliced into 1 cm intervals from 3 cm down to the bottom (ranging from 11–19 cm, depending on the core) to analyze the vertical distribution of MPs within the core. The samples were stored in 500 mL glass jars at −20 °C until further analysis. Samples from Cores 2 and 3 were preserved for further research. Core 4 was completely sealed from air and immediately frozen on board (−20 °C) for total organic carbon (TOC) and radiometric dating analyses.

### 2.2 Lithology and Age Model

Color photographs of sediment samples from one of the PVC cores were captured on the deck of the vessel at each station to document the core’s lithology (Figure S2 and Paragraph S1). In the laboratory, Core 4 of each station was sliced while frozen (Figure S3), following the same top 3 cm and then 1 cm intervals as Core 1. Each sample was divided into two halves. One half was used to determine TOC, while the other half was used for radiometric dating.

Sample preparation for radiometric dating was carried out as described by Bunzel et al.^37^ and Logemann et al.^38^ In brief, each sample underwent weighing and drying at 110 °C until a constant weight was achieved. Subsequently, the wet density (W.D., g cm^–1^), water content, porosity, and dry bulk density (DBD, g cm^–1^) were determined (Table S1). The dry sediment was homogenized using a ball mill (300 rpm, 15 min, PM 400 Retsch, Germany). Approximately 10–26 g of the sample material was then sealed in a gastight Petri dish and stored for a minimum of 28 days to ensure equilibrium conditions between ^226^Ra and its product isotopes ^222^Rn, ^214^Pb, and ^214^Bi. The quantification of ^210^Pb, ^137^Cs, ^214^Pb, and ^214^Bi was carried out using high-purity low-level germanium detectors (BE 3830P-7500SL-ULB, GX-3018, Mirion Technologies (Canberra), Germany) (Paragraph S2). For calibration, an artificial reference material was prepared with silica gel and reference solutions of ^137^Cs and ^226^Ra (Eckert & Ziegler Nuclitec GmbH, Germany). Measurement times ranged between 90,000–600,000 s, depending on the sample activity.

The age model of the sediment cores is based on the tephrochronology complemented by ^210^Pb/^137^Cs dating. The age of the sediments was calculated with a correction for the increasing compaction with depth in the upper sediments.^39^ For More details please refer to Paragraph S3.

### 2.3 Total Organic Carbon Measurements

TOC was measured at the University of Bremen (Germany) by combustion with a CHN Analyzer (HERAEUS) according to the method described by Romero et al.^40^ For this, samples were dried overnight at 60 °C, weighted and treated with 2N hydrochloric acid (HCl) to remove carbonates. Based on comparison with internal lab standards, the overall precision was better than 0.1%.

### 2.4 Microplastic Analyses

#### 2.4.1 Microplastic Extraction

At each station, samples of both the overlying water and sediment in Core 1 were analyzed for MPs. Before MP identification, several preparatory steps were required to isolate the MP fraction, including density separation and organic matter digestion. For sediment samples, initially, each sample was thawed and dried at 60 °C until reaching a constant weight. Before drying, all samples were covered with perforated aluminum foil. Once dried, the sediment was gently homogenized in the glass jar using a metal spoon. Approximately 35 g of dried sediment from each sample was transferred into a 150 mL glass beaker (Table S1). Then, 100 mL of prefiltered (Paragraph S4) sodium bromide solution (NaBr, Gruessing GmbH, Germany, density = 1.53–1.55 g cm^–3^,^41^) was added to the beaker and left overnight to ensure complete rehydration of the sample. If the sample weighed less than 35 g, the entire sample was analyzed. The samples were then processed following a multistep extraction protocol. For full workflow details, please refer to Paragraph S5. In brief, the procedure consisted of three main steps: (1) First density separation of rehydrated samples using prefiltered NaBr, transferring lighter materials from the samples onto 10 μm stainless steel meshes (Ø 47 mm; HAVER & BOECKER OHG, Germany). (2) Oxidation (Fenton’s treatment; according to Al-Azzawi et al.^42^ with minor modifications) with iron sulfate (FeSO_4_, 20 g L^–1^, AppliChem GmbH, Germany) and hydrogen peroxide (H_2_O_2_, 30%, Fa. Bernd Kraft GmbH, Germany) for 20 min to digest organic materials. This was followed by the slow addition of 4 mL of 97% sulfuric acid (H_2_SO_4_) and 10 mL of Tween 20 (0.1%, VWR International, France) to remove precipitated iron formed during the reaction and to prevent particles from adhering to the glass wall, respectively. The treated samples were then concentrated on new 10 μm stainless steel meshes. (3) Second density separation using prefiltered NaBr to remove further inorganic residues and concentration of samples onto new small 10 μm meshes. The filters were then flushed with 500 mL Milli-Q water (Milli-Q, IQ 7000, Millipore, France) to remove any NaBr residues. Following that, the materials on the filter were carefully rinsed with Milli-Q water and retained in a glass wide-neck bottle (100 mL), stored at 4 °C for later analysis. Overlying water samples were thawed and filtered onto 10 μm stainless steel meshes, followed by Fenton’s treatment and density separation, following the procedures described above.

For MP identification, the sample material was concentrated on aluminum oxide filters (Ø 25 mm; 0.2 μm pore size; Anodisc, Whatman, U.K.). Depending on the residual material load in the processed samples, one to three Anodisc filters were prepared per sample. The Anodisc filters were then stored in glass Petri dishes (Ø 6 cm) and dried for at least 24 h in a desiccator (Sicco, Bohlender GmbH, Germany) before analysis by micro-Fourier transform infrared spectroscopy (μFTIR).

#### 2.4.2 Microplastic Identification

Putative MPs concentrated on the Anodisc filters were measured by a μFTIR-microscope (Hyperion 3000) connected to a Tensor 27 spectrometer (Bruker Optik GmbH, Germany) equipped with a 3.5× objective and a 64 × 64 focal plane array (FPA) detector with a pixel size of 11 μm, which sets the lower detection limit of the present analysis. A spectral range of 1250–3600 cm^–1^ with 32 coadded scans collected at a resolution of 8 cm^–1^ was used.^19,43^ A grid of 20–26 measurement fields was applied to cover all particles in the filtration area. Due to the presence of coal particles and shell residues in certain samples (Figure S4), a barium fluoride (BaF_2_) window was not used to cover the Anodisc filters. Consequently, the MP particles were not morphologically categorized into elongated particles (fiber-like MPs with an aspect ratio of 3:1 or higher)^44^ and particle-like MPs. The IR spectra obtained were processed with OPUS 8.8 software. Subsequently, automatic identification and quantification of MPs were conducted using an updated version of siMPle,^45,46^ utilizing a reference database originally designed by Primpke et al.^47^ and updated by Roscher et al.^48^ The reliability of automatic polymer identification, within a 95% confidence interval and the strategy of classifying polymer clusters, is detailed in Primpke et al.^47^ The final tabular data, including the size of each MP particle and the specified polymer clusters, were obtained directly from the siMPle spectra analysis (version 1.3.2.1, available upon request, more details see Paragraph S6).

### 2.5 Quality Assurance and Quality Control

Several measures were implemented to minimize the potential contamination of samples with MP particles. All details are reported in Paragraph S4.

### 2.6 Statistical Analyses

**Figure 2:**
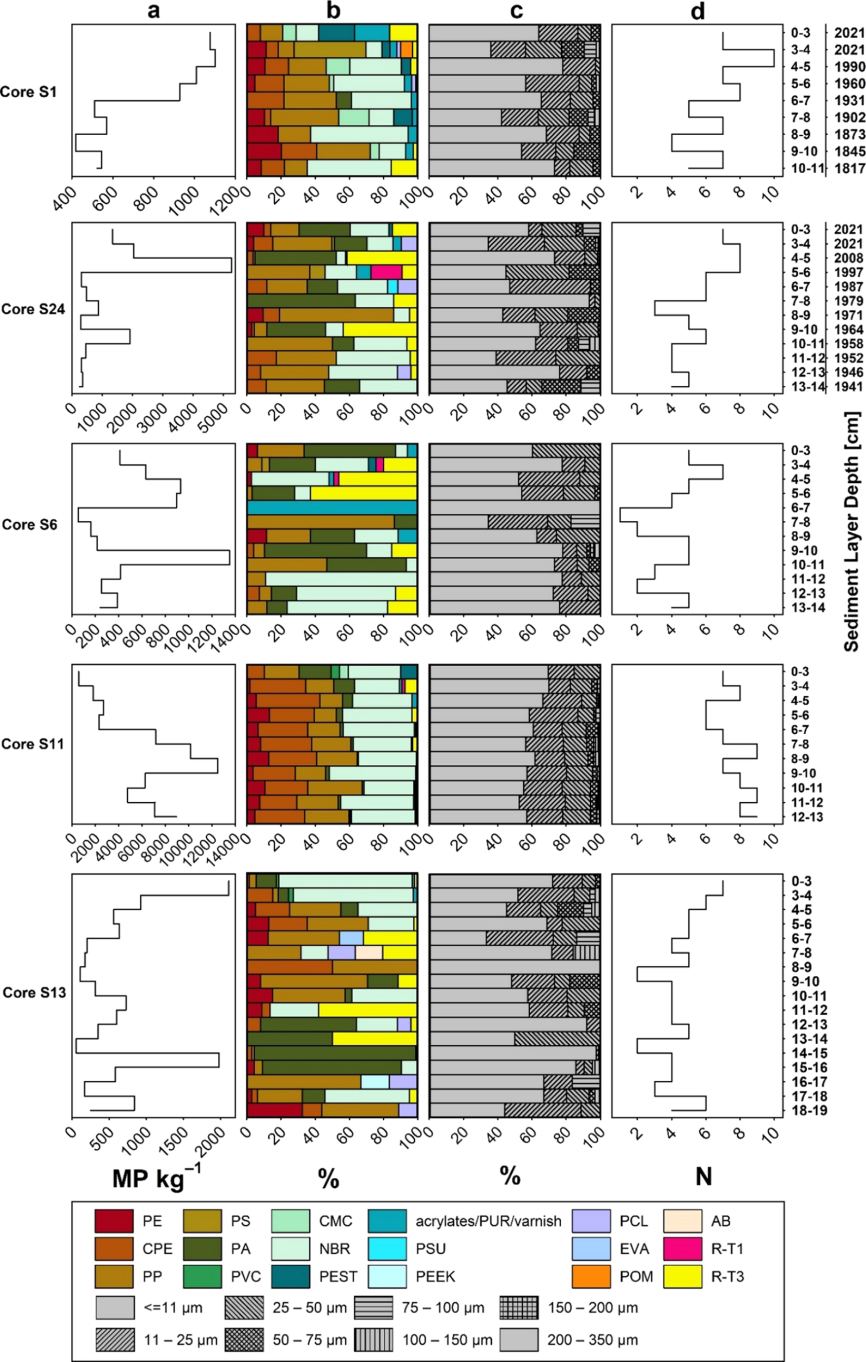
Vertical distribution of microplastics in each sediment layer of each core. (a) Concentrations of microplastic particles (MP kg^–1^), to better illustrate the variation of MP concentrations with depth, we opted to use absolute numbers instead of logarithmic data. (b) Polymer composition. (c) Size class distribution. (d) Polymer richness (N). Steps indicate the sampling horizons for each core. Sediment layer depth and corresponding age is given. polyethylene (PE), chlorinated polyethylene (CPE), polypropylene (PP), polystyrene (PS), polyamide (PA), poly(vinyl chloride) (PVC), chemically modified cellulose (CMC), nitrile rubber (NBR), polyester (PEST), polyurethane (PUR), polysulfone (PSU), polyether ether ketone (PEEK), polycaprolactone (PCL), ethylene vinyl acetate (EVA), polyoxymethylene (POM), acrylonitrile butadiene (AB), rubber type 1 (R-T1), rubber type 3 (R-T3).

The amounts of MPs determined in sediment and overlying water samples were blank-corrected by subtracting the average amounts found in procedural blanks, respectively (Table S2). All aliquots per sample were summed up for analysis. For sediment, the particle count [n (MP) kg^–1^] was calculated based on the dry sediment weight. To better illustrate the variation of MP concentrations in sediments with depth, we opted to use absolute numbers instead of logarithmic data (Figure 2a). For overlying water, the particle count [n (MP) L^–1^] was calculated based on the sample volume. To assess the polymer diversity, species richness (N) was calculated. The relationships between MP concentrations, smallest detectable size class (11 μm) percentage, polymer diversity, and ancillary data (depth, TOC, W.D., DBD, and porosity) were tested using Spearman’s Rank Correlation due to the non-normally distributed nature of our data (Statistica 14.1.0, StatSoft GmbH, Germany). Maps showing the geographical location of the samples were produced using QGIS 3.26.3 with the base map ESRI Ocean (QGIS Development Team). Graphs were created in SigmaPlot 13.0 (Systat Software Inc.).

## 3 Results and Discussion

### 3.1 Sediment Archive

**Figure 3:**
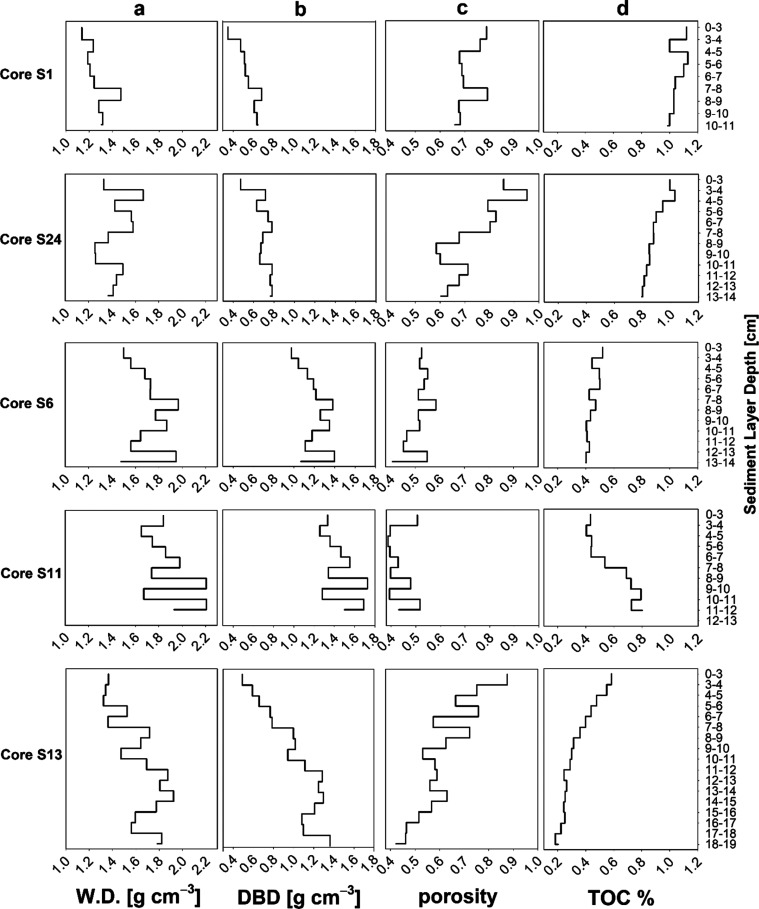
Vertical profiles of ancillary data in sediment cores. (a) Wet density (W.D., g cm^–3^). (b) Dry bulk density (DBD, g cm^–3^). (c) Porosity. (d) Content of total organic carbon (TOC).

The combination of ^210^Pb and age-depth modeling techniques,^39^ analyzed with 1 cm resolution, provided reliable chronologies for two out of the five investigated sediment cores: cores S1 and S24 collected from the Fedje/Shetland transect. At these stations, the subcores that were collected 10 cm apart from each other, contained similar changes in lithologic characteristics and color changes over depth, which indicates that sediment deposition was similar at the subcore coring positions. The uppermost layer (0–3 cm) was excluded from age estimation due to the assumption of bioturbation. This assumption was further supported by lithological observations, as the sediments collected along the Fedje/Shetland transect (S1, S24, and S6) consisted of heavily bioturbated mud and clay-rich sand. According to the age model, the ages of the top 0–3 cm and 3–4 cm layers of Cores S1 and S24 were assumed to correspond to the year 2021, reflecting the time of sampling activities. Although lower parts of core S1 were below the dating horizon of the ^210^Pb method, no large change in sediment composition and particle size was observed. Furthermore, no perturbations in sediment structure were observed. This suggests a constant sediment deposition and bioturbation throughout the core and allows us to extrapolate the age to deeper layers (Paragraph S3). The oldest sediment layer (10–11 cm from core S1) was estimated to date back to approximately 1817 (Figure 3d). In Core S24, the oldest sediment layer (13–14 cm) was estimated to date back to the 1940s. Detailed vertical profiles of unsupported ^210^Pb and ^137^Cs activities for each core are provided in Figure S6.

Age estimation for core S6 was not feasible due to signs of disturbance attributed to fisheries activity. This resulted in the mixing of recent and subrecent materials, predominantly near the surface, as indicated by the unsupported ^210^Pb activity (Table S1 and Figure S6). The same applies to core S11, collected near Bear Island. Given the long tradition of bottom trawling on the continental shelf in Norwegian waters,^49^ these results were not unexpected.

Lithological observations of core S11 revealed stiff, dark-gray clays at the base, transitioning into a layer of dark-gray clay mixed with yellow sandy clay. This transition zone was overlaid by a layer of yellow sandy clay containing individual pebbles of various sizes, which were rounded but not sorted. There were no signs of bioturbation within the sediment. Examination of the palynological content revealed that the clay was of terrestrial origin, containing triplets and monolete spores without marine palynomorphs. In contrast, the overlaying sandy clays had a marine origin and contained microfossils such as organic-walled dinoflagellate cysts, and benthic- and planktic foraminifera.

Core S13, collected from the Fugloya Bjornoya transect, displayed a gradual transition from gray clays to yellow sandy clays containing randomly distributed pebbles. Dating proved challenging due to recent sediment deposition observed in the upper centimeter. Additionally, lithological observations indicated the intermixing of materials from other locations throughout the deeper sections of the core.

### 3.2 Microplastics in Water-Sediment Interface

High concentrations of MPs were detected in all overlying water samples from the five sediment cores, ranging from 3–25 MP L^–1^ (Figure 1b). The highest concentrations were found at stations within the Arctic Circle, S11 and S13, with 25 and 23 MP L^–1^ detected, respectively. This was followed by S24 (20 MP L^–1^), S6 (12 MP L^–1^), and S1 (3 MP L^–1^) from the Fedje/Shetland transect. Compared to the associated 0–3 cm sediment layer (Figure 2d), the polymer diversity in the overlying water was significantly lower. Only four polymer types were identified by μFTIR imaging, with S24 exhibiting the highest polymer diversity (*n* = 4, Figure 1c). Polyamide (PA), polyester (PEST), acrylates/polyurethane (PUR)/varnish, and rubber type 3 (R-T3, ethylene propylene diene monomer) each accounted for a quarter of the polymer diversity. In S6, PA and R-T3 were identified, accounting for 14.3 and 85.7%, respectively. In the overlying water samples from S1, S11, and S13, PA was the only polymer identified (Figure 1c). Unlike previous studies investigating small MPs in different water depths in the study area,^50^ where R-T3 was excluded due to lipid misassignment, manual spectra inspection in this study indicated no such errors (details see Figure S7).

Only a few studies have investigated MP concentrations at the water-sediment interface in the marine environment.^19,51^ The use of different sampling and analysis methods makes it challenging to directly compare findings across studies. Martin et al.^51^ siphoned the water-sediment interphase from the Irish Continental Shelf and analyzed MPs down to 250 μm, reporting the highest concentrations of MPs accumulating at the water-sediment interface and top 0.5 cm of sediments. In contrast, our study analyzed MPs down to 11 μm, with the largest size class identified being 175–200 μm (Figure S8). Compared to samples collected from the surface, subsurface, and deeper waters at the same station,^50^ MP concentrations in the overlying water were found to be up to 10^4^ times higher, especially at stations within the Arctic Circle (S11 and S13). It remains unclear to what extent this significant difference can be attributed to the disparity in collected sample volume. Surface and deeper waters are relatively easy to obtain, with volumes ranging between 0.3 × 10^3^ to 1.6 × 10^3^ L.^50^ However, collecting large volumes of overlying water in sediment cores is not feasible due to the limitation of the applied sampling device (MUC).

Despite the limitations imposed by the small sample volumes, our results still provide a unique insight into the distribution of MPs in the overlying water in intact sediment cores. High concentrations of MPs in the water-sediment interface might increase their accessibility and pose significant risks for benthic organisms such as filter feeders.^52,53^ These particles could originate from the complex biological and physical sinking process from the ocean surface^30,54,55^ or result from resuspension from the sediment.

### 3.3 Microplastic Vertical Profiles in Sediment Cores

#### 3.3.1 Microplastic Concentrations and Polymer Diversities

MP concentrations in each sediment layer from all cores collected in the NCC varied strongly (54–12,491 MP kg^–1^) (Figure 2a). The highest concentrations were detected at S11 within the 6–13 cm layers (4750 MP kg^–1^ (10–11 cm layer) −12,491 MP kg^–1^ (8–9 cm layer), Figure 2a, Station 11), where the sediment was collected in the Arctic Circle near Bear Island (Figure 1a). Additionally, layer 4–5 cm from S24 also exhibited a relatively high MP concentration (5270 MP kg^–1^, Figure 2a, Station 24). The lowest concentration was found in the 6–7 cm layer (54 MP kg^–1^) from S6, which was collected from the Fedje/Shetland transect. MPs in the top 3 cm of the five cores ranged from 412–2111 MP kg^–1^ (Figure 2a). Our results are comparable to the surface sediments collected at the HAUSGARTEN observatory in the Arctic (west of Svalbard, top 5 cm, 42–6595 MP kg^–1^)^27^ and in the western Arctic Ocean (top 2 cm, 331–1369 MP kg^–1^).^32^

A total of 18 different polymer types were identified across all the sediment layers (spectra details see Figure S9), with polymer diversities varying from one (layer 6–7 cm of core S6) to 10 (layer 3–4 cm of core S1) (Figure 2d). For samples collected at the Fedje/Shetland transect, the 3–4 cm layers exhibited the highest polymer diversity (*n* = 7–10) (Figure 2d), exceeding that of the top 3 cm (*n* = 5–7). Surface sediment resuspension could be an explanation. The distribution of polymer diversities varied between sediment cores. In cores S1, S6, and S13, there was generally no discernible trend indicating a decrease in polymer diversity with increasing sediment depth. In core S24, a significant negative correlation between polymer diversity and depth was observed, whereas core S11 showed the opposite. In all sediment cores, polymers identified in surface sediments were also present in deeper layers. The polymer compositions of each layer are depicted in Figure 2b, with polyethylene (PE), chlorinated polyethylene (CPE), polypropylene (PP), PA, nitrile rubber (NBR), acrylates/PUR/varnish, and R-T3 being present in all sediment cores collected. In terms of polymer contribution, in each core, NBR and PP contributed on average between 22.1–36.5 and 19.5–28.8%, respectively (Figure S10). In cores S24, S6, and S13, PA contributed on average 20.3 to 23.1%, while in core S11, CPE followed NBR, contributing 26.7% (Figure S10). The other polymers contributing less than 5% in different sediment cores were also identified, including polystyrene (PS), PVC, chemically modified cellulose (CMC), PEST, polysulfone (PSU), polyether ether ketone (PEEK), polycaprolactone (PCL), ethylene vinyl acetate (EVA), polyoxymethylene (POM), acrylonitrile butadiene (AB) and rubber type 1 (R-T1, a mixture of natural rubber, fillers and synthetic rubber^47^) (Figure S10).

The predominant presence of PP and CPE in the sediment cores is not unexpected, as they are the most widely used polymers in Europe.^13^ These findings align with our observations from surface water samples.^50^ Moreover, widespread distributions of PP and PE have been consistently reported across various environmental matrices across the North Atlantic, Barents Sea, and the Arctic.^20,28,32,56^ Interestingly, discussions have arisen regarding the absence of PA from the sea surface.^51,57^ PA is a crucial material used in fishing gear.^58^ Given the NCC’s extensive history of intense fishing activities over decades,^49^ the significant proportion of PA identified in our study further strengthens the hypothesis that the deep sea serves as a significant accumulation zone for this type of plastic. In our study, another significant contributor is NBR, known for its exceptional resistance to various temperatures, as well as to substances like oil, gasoline, and chemicals.^59^ It finds extensive applications, including hoses, seals, O-rings, or transmission belts in offshore oil platforms. Similarly, high proportions of NBR were also identified in the snow, sea ice cores, and deep-sea sediment collected in the Arctic.^20,27,59^ However, it is noteworthy that we observed a few NBR spectra that may have been misassigned from shell residues on the Anodisc filters (Figure S11), potentially leading to an overestimation of the actual concentrations of NBR. Despite the occasional presence of shells in sediment samples, a subsequent random selection of NBR spectra demonstrated satisfactory matches. Pyrolysis gas chromatography–mass spectrometry (py-GCMS) covalidation further supports our decision to retain this polymer in the results (unpublished data). However, further improvement of the database is needed to enhance accuracy, particularly regarding the clusters that may be easily misassigned from natural materials.

#### 3.3.2 Size Distribution

Across most layers (97%), the size distribution was skewed toward the smallest size class (34–100%, Figure 2c). Our findings are consistent with those of other sediment studies employing the same analytical method.^19,60,61^ In Core S11, collected near Bear Island, numerous black particles resembling coal were observed on the Anodisc filters (Figure S4a). This posed a challenge to using a BaF_2_ window to cover the Anodisc filters during FPA-μFTIR measurements. Similar observations were also noted in sediment samples collected in the deep sea west of Svalbard.^27^ In addition, in sediments collected from other locations in our study, a few shell residues were still present on the filters (mostly foraminifera, Figure S4b). Consequently, we were unable to morphologically categorize elongated particles and particle-like MPs in the sediment samples. Extraction methods for MPs need further improvement, especially when analyzing sediments containing a variety of substances.

#### 3.3.3 Microplastic Distribution in Time and Space

Previous studies have focused on MP contamination in surface sediments due to its tendency to accumulate mainly in surface sediments.^62^ Numerous studies have reported the accumulation of MPs in surface sediment (top 5 cm), spanning from freshwater environments such as rivers and lakes to marine ecosystems, extending from coastal regions to the hadal trench, and from the equator to the poles.^19,27,61,63−65^ However, scientists have recently shifted attention to MP distribution in deeper sediment layers as they may also be preserved there. Some studies have reported varying MP concentrations with depth in vertical sediment profiles and discussed using MPs as an indicator for the Anthropocene.^32,66−69^ In this study, MPs were found not only in surface layers (top 5 cm) of sediments but also in deeper layers, reaching depths of up to 19 cm. Remarkably, MPs seem to have traveled through time, leaving traces in sediments predating the 1930s and 1940s, before plastics became increasingly prevalent in the consumer marketplace.^70^

Figure 2 illustrates the vertical profiles of MP concentration, polymer compositions, size distributions, and polymer diversities across sediment cores collected from various locations in the NCC. In core S1, which age was successfully estimated, the highest MP concentration was identified in the 3–4 cm layer (1102 MP kg^–1^), followed by the top 0–3 cm (1076 MP kg^–1^) and 4–5 cm layers (1010 MP kg^–1^). These layers were estimated to span between 1990 and 2021 (Figure 2d, Station 1). The following 5–6 cm layer has an MP concentration of 928 MP kg^–1^ and an age span between 1960 to 1990. Contrary to our hypothesis, we did not observe a significant negative trend between MP concentrations and increasing depth in the post-1950 layers (top 6 cm, Table S3). These results stand in contrast to studies reporting an exponential increase in plastic burial rate over several decades.^32,67,69,71^ One factor that may affect such results is bioturbation, as indicated by the lithology (Figure S2). Core S1 comprises heavily bioturbated mud with a low abundance of foraminifera. The surface of core S1 exhibits several burrows with branched structures, each a few millimeters in diameter, possibly attributed to benthic organisms activities. For example, lugworms are known to reside even up to 70 cm below the sediment surface.^57^ Their activity in sediment reworking could alter sediment stratigraphy post-MP deposition. Consequently, the upper sediment layers containing MPs might undergo partial or complete homogenization, which could compromise the accurate temporal record and potentially result in the downward movement of MPs.^7,57^ Additionally, our study used a relatively low resolution for age estimation compared to Courtene-Jones et al.^71^ who sliced the top 5 cm sediment into 0.5 cm sections and employed 1 cm intervals between 5–10 cm. This difference may introduce bias into our results, as we homogenized the top 3 cm for MP analysis.

However, unlike the post-1950 layers, when observing the full vertical profiles in this core (S1), we noticed a significant negative correlation between MP concentrations and increasing depth (extending until 11 cm) (Table S4). Surprisingly, MPs were detected in all deeper layers dating back to 1931, including a layer dating back to 1817, with a concentration of 523 MP kg^–1^. This finding indicates the presence of modern plastics (Figure 2b, station 1). The first synthetic polymer was only invented in 1907^10^ and the bulk of plastic production occurred since the 1950s.^12^ Additionally, it can be assumed that it takes several decades for certain MP polymers to be prevalent in the environment following their mass production. This is supported by pioneering studies from the early 1970s, which reported significant amounts of plastic particles in neuston samples over large geographic areas of the North Atlantic.^72,73^ Polymers such as PA, PS, PVC, and PE began to be manufactured in the late 1930s and 1940s.^5^ Therefore, theoretically, these polymers are not anticipated to be detected in environmental samples predating their invention or commercialization. However, our observation of traces of burrows in the upper part of the core indicated that bioturbation has affected the sediments, automatically transporting MPs to the deeper layers of the core. This conclusion is further supported by the detection of low concentrations of ^137^Cs in the deeper layers of the core (Figure S6), which, according to the age model, were deposited well before the 1960s, the time of the first worldwide deposition of ^137^Cs. Dimante-Deimantovica et al.^6^ also discovered MPs in sediment layers dating back to 1733 collected from lakes in northeastern Europe. Similarly, Xue et al.^62^ found MPs in the bottom of a core (−60 cm) collected from the Northwest Pacific Ocean, with a computed date of 1897. Furthermore, Courtene-Jones et al.^71^ also reported the presence of MPs in all sediment layers predating the 1940s (4–10 cm) collected from the Rockall Trough, North Atlantic Ocean. These findings suggest that MPs may persist in sediment layers deeper than previously thought. Several mechanisms have been discussed in these studies. For example, Courtene-Jones et al.^71^ revealed a significant positive correlation between MP concentrations and sediment porosity, indicating potential redistribution of MPs within pore waters. In our study, although no correlations were found between MP concentrations and porosity in core S1 (Table S4), the porosity ranged from 0.66 to 0.79. This is notably higher than the porosity in the deeper layers of the sediment core (below 0.65) collected by Courtene-Jones et al.^71^ Additionally, an irregularly high porosity was observed at a depth of 7–8 cm in core S1, which is comparable to the surface sediments (Figure 3c, station 1), potentially facilitating the passage of MPs. Thus, besides bioturbation, porosity may be another factor influencing the downward transport of MPs, although the exact mechanism is not yet fully understood.^32,71^

Some studies propose MPs in sediment layers predating the 1950s stem from contamination. For example, Brandon et al.^69^ considered fibers found in pre-1945 layers in a minimally bioturbated core collected near an urban area in California, USA to be indicative of procedural contamination. In our study, all samples were analyzed alongside procedural blanks and the results were blank-corrected accordingly. Given the low background contamination on board and in our laboratory, and considering that the core material was stainless-steel metal, we are skeptical about attributing all MPs present in the pre-1950 layers solely to contamination. Moreover, the use of FPA-μFTIR and automatic data analysis of small MPs down to 11 μm reduced observer bias compared to visual inspection methods.^46,74^ As shown in Figure 2c, all MPs identified in our study were below 350 μm, with the smallest size class (11 μm) predominating most sediment layers (97%) with relative abundances ranging from 34 to 100%. It is noteworthy that other studies reporting the vertical distribution of MPs in sediment cores employed visual inspection combined with Fourier transform infrared (FTIR) spectroscopy methods, with a minimum size class of 60 μm.^51,62,69,71^ It has been revealed that MP particle size significantly affects penetration profiles, as MPs with smaller sizes exhibit greater mobility: MPs with a size range of 10–20 μm could penetrate deeper compared to those with sizes of 100–150 and 300–450 μm.^75^ Thus, the use of a higher minimum detected size class could potentially result in a significant underestimation of MP concentrations in deeper sediment layers.

In contrast to core S1, core S24, which was collected from the same sampling transect (Fedje/Shetland transect) and also had its age successfully estimated, displayed a clear declining trend of MP concentrations with increasing depth, from recent times back to the 1940s, which is aligned with the results from other studies.^32,51,67^ Unlike core S1, this core (S24) shows no burrows of tube worms and only minor signs of bioturbation, suggesting that extensive bioturbation did not occur over large depths. This was further supported by the ^137^Cs observation (Figure S6), which according to our age model, was not present in sediment layers deposited before the 1960s. The highest MP concentration in this core was found in the 4–5 cm layer (5270 MP kg^–1^), which was estimated to span from 2008 to 2021. This is not surprising when considering the timeline of global plastic production.^12^ Following this are the 3–4 cm and top 0–3 cm layers, with 2037 and 1343 MP kg^–1^ detected, respectively. The lower concentrations in the uppermost layers (0–2 cm) compared to the following layers (2–4 cm) were also documented by Xue et al.^62^ This could be attributed to the dynamic instability at the surface layer interface, resulting in the resuspension of MPs. The high concentrations of MPs in the overlying water further support this explanation (Figure 1b). Interestingly, a peak in MP concentration was observed in the 9–10 cm layer, spanning from the year 1964 to 1971 (Figure 2d, station 24), with a concentration of 1913 MP kg^–1^, which is comparable to the top 3–4 cm layer. This phenomenon aligns with similar observations in a sediment core collected from an urban lake in the U.K.^76^ Kim et al.^32^ also demonstrated an increasing trend of MP burial rate from the year 1960 to 1970 in the sediment core collected from the western Arctic Ocean.

As mentioned in Section 3.1, dating cores S6, S11, and S13 posed challenges. When analyzing the vertical profiles of MP concentrations in core S6, we observed a more random pattern. The highest concentration was found at 9–10 cm (1352 MP kg^–1^), while the lowest was found at 6–7 cm (54 MP kg^–1^) (Figure 2a, station 6). The age of this core cannot be accurately predicted due to the physical disturbance indicated by the ^210^Pb data (Figure S6), likely resulting from activities such as fishing. This may also explain the relatively erratic distribution of MP concentrations in the core. While no significant correlations were observed between MP concentrations and increasing depth in this core, a contrasting result was found in core S11. A significant positive correlation between MP concentration and depth was observed. Notably, in core S11, the deeper layers (6–13 cm layers) exhibited the highest MP concentrations among all sediment layers collected, ranging from (10–11 cm) with 4750 MP kg^–1^ to (8–9 cm) with 12 491 MP kg^–1^ (Figure 2a, station 11). Unexpectedly, the lowest MP concentration was found at the top 0–3 cm, with 584 MP kg^–1^ detected. However, due to fishing disturbance of this core, the reason for this phenomenon remains elusive, suggesting the possibility of a random result. In Core S13, which cannot be dated due to the low sedimentation rate, the highest MP concentration was found at the top 0–3 cm layer (2111 MP kg^–1^).

Overall, core S11, collected in the Arctic Circle near Bear Island, exhibited the highest MP concentration compared to the other four cores obtained along the NCC. Bear Island, located in the Barents Sea, experiences the influence of strong ocean currents, including the NCC, which has the potential to transport plastic waste from regions in the North Atlantic where plastic pollution is more prevalent.^34,77−79^ Comparable concentrations were also observed in Arctic deep-sea sediment.^27,28^ The presence of high concentrations of MPs along the Bjørnøya transect in the Barents Sea suggests that this area may be situated near or within a plastic accumulation zone.

#### 3.3.4 Ancillary Data

Ancillary data for each sediment core is presented in Figure 3. Spearman’s Rank Correlations of MP concentrations, polymer diversities, and the percentage of the smallest size class (11 μm) with the respective ancillary data of each sediment core are provided in Table S4. In cores S6 and S13, no correlations were observed between MP concentrations and ancillary data, which include depth, wet density (W.D.), dry bulk density (DBD), porosity, and TOC. However, in core S1, a negative correlation was found between MP concentrations and DBD (ρ = −0.68, *p* < 0.05). Interestingly, in cores S24 (ρ = 0.71, *p* < 0.05) and S11 (ρ = 0.66, *p* < 0.05), positive correlations were identified between MP concentrations and TOC. In cores S1, S24, and S13, unaffected by fishing, only core S1 showed a significant negative correlation between the smallest size class percentage and porosity (ρ = −0.71, *p* < 0.05). Conversely, in cores S6 and S11, impacted by fishing, significant negative correlations are observed between the smallest size class percentage and TOC. The influence of anthropogenic activities (e.g., fishing) on these two sediment cores prevents us from providing meaningful explanations regarding the environmental data and the vertical distribution of MPs. These results highlight the considerable variability in sediment core characteristics across different sampling sites in the NCC. Moreover, additional studies reporting correlations between MP concentrations and environmental variables have shown inconsistent results. For example, in surface sediment collected from the western Arctic Ocean, no correlations were observed between MP concentrations and porosity or TOC.^32^ However, contradictory findings have been reported in other studies.^71^ The heterogeneity of seafloor topography likely plays a significant role in shaping the vertical distribution of MPs. These disparities suggest that environmental factors may not be as reliable in predicting MP accumulation zones in sediments.

This study provides an extensive examination of the vertical distribution of MPs in sediment cores retrieved from European waters to the Arctic waters along the NCC. Our findings unveil considerable variability in MP concentrations within the study area and underscore the widespread presence of MPs throughout the sediment cores, predating the advent of plastics. The elevated concentrations of MPs observed in sediment near Bear Island indicate a possible accumulation zone for MPs in this area. Furthermore, this study discussed potential mechanisms of the downward transportation of MPs within the sediment core, including factors such as bioturbation, pore water dynamics, and polymer sizes. Due to the current lack of standardized MP sampling and analytical methods, differences in research methodology make comparisons between studies difficult. In addition, the heterogeneity of seafloor topography and the impact of anthropogenic activities (e.g., fishing) results in varying environmental factors from one station to another, which may also contribute to differences in the vertical distribution of MPs. This casts doubt on the reliability of using environmental parameters to predict potential MP accumulation zones and using MPs in sediment cores as an indicator for the Anthropocene.

## Supporting Information Available

Procedural blanks in overlying water and sediment samples, Spearman’s rank correlation of MP concentrations, smallest size class percentage, polymer diversity and ancillary data in post-1950s layers in station 1, and all layers from all sediment cores (Tables S1–S4); images of multiple corer equipped with a combination of eight PVC tubes and four metal tubes, lithology observations of the sediment cores, core slicing while frozen in the lab, coal and shell residues on Anodisc filters, reference materials, vertical profiles of unsupported ^210^Pb and ^137^Cs activities in all sediment cores, visual image and image analysis of sediment-water phase of station 6, percentage of each size class in overlying waters of different sediment cores, spectra of polymers detected in all sediment cores, mean percentage of each polymer type in each sediment core, good and bad quality nitrile rubber spectra (Figures S1–S11); lithology description of the sediment cores, details of radiometric dating method, description of age model calculation, quality assurance and quality control, workflow of extraction of MPs from sediment samples, details for MP identification (Paragraphs S1–S6) (PDF)
Geographical location of sampling points, concentrations of each polymer, MP concentration of each sample, sampling depths, sediment dry weight used for analysis, polymer diversity, TOC%, wet density, water content, porosity, dry bulk density, estimated age of core S1 and S24, unsupported ^210^Pb and ^137^Cs activities (Table S1) (XLSX)
