# Supplementary material for: Diving into the Depths: Uncovering Microplastics in Norwegian Coastal Sediment Cores
Source: Environ Sci Technol. 2024 Sep 11;58(38):17036–47. doi: 10.1021/acs.est.4c04360 (PMC11428159; doi:10.1021/acs.est.4c04360)
Supplement: Supplementary file 1 — es4c04360_si_001.pdf [file es4c04360_si_001.pdf]

Supporting Information

Diving into the Depths: Uncovering Microplastics in Norwegian Coastal Sediment Cores

Fangzhu Wu[a,*], Karin A.F. Zonneveld[b,c], Hendrik Wolschke[d], Robin von Elm[a], Sebastian Primpke[a], Gerard J. M. Versteegh[b, e], Gunnar Gerdts[a]

[a]Alfred-Wegener-Institut Helmholtz-Zentrum für Polar- und Meeresforschung, Biologische Anstalt Helgoland, Kurpromenade 201, 27498 Helgoland, Germany

[b] MARUM - Centre for Marine Environmental Sciences, University of Bremen, 28359 Bremen, Germany

[c] Department of Geosciences, University of Bremen, 28359 Bremen, Germany

[d] Environmental Radiochemistry, Institute of Coastal Environmental Chemistry, Helmholtz-Zentrum Hereon, 21502 Geesthacht, Germany

[e] Department of Physics and Earth Sciences, Constructor University, 28759 Bremen, Germany

*Email: fangzhu.wu@awi.de

Number of Figures: 11

Number of Tables: 4

Number of Paragraphs: 6

Number of Pages: 28

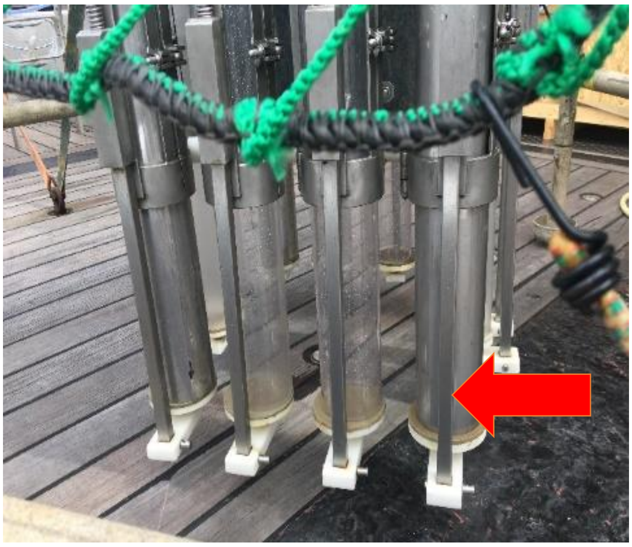

Figure S1. Multiple corer (MUC) equipped with a combination of eight polyvinyl chloride tubes (PVC) in the middle and four stainless steel metal tubes at the corners.

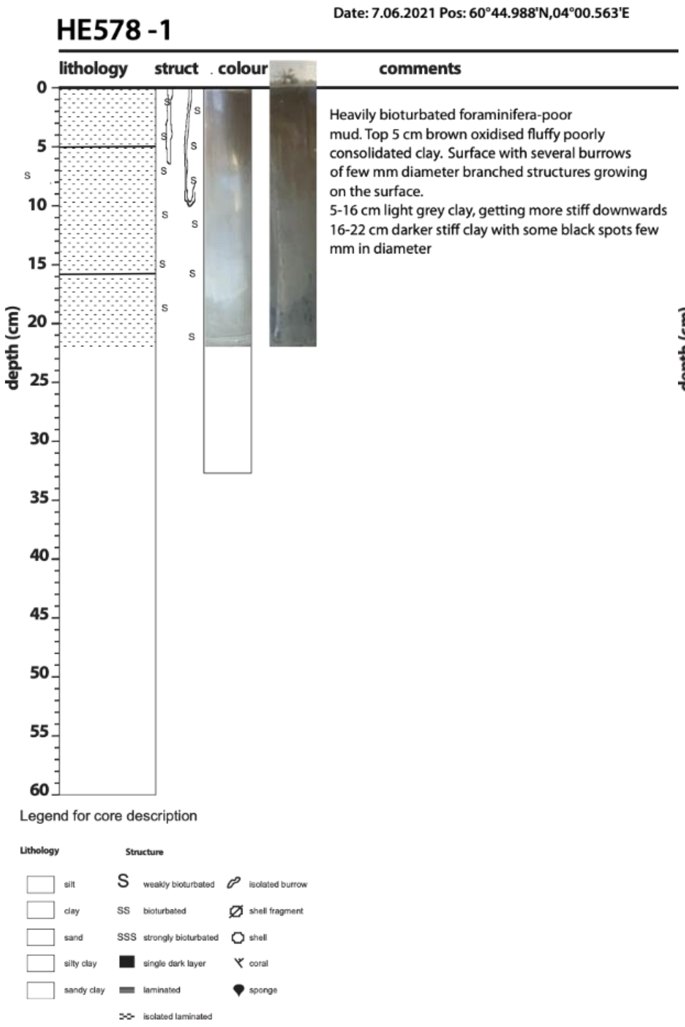
HE578 -1
Date: 7.06.2021 Pos: 60°44.988'N,04°00.563'E
lithology
struct
colour
comments
depth (cm)
Heavily bioturbated foraminifera-poor mud. Top 5 cm brown oxidised fluffy poorly consolidated clay. Surface with several burrows of few mm diameter branched structures growing on the surface.
5-16 cm light grey clay, getting more stiff downwards
16-22 cm darker stiff clay with some black spots few mm in diameter
Legend for core description
Lithology
Structure
silt
clay
sand
silty clay
sandy clay
weakly bioturbated
bioturbated
strongly bioturbated
single dark layer
laminated
isolated laminated
isolated burrow
shell fragment
shell
coral
sponge

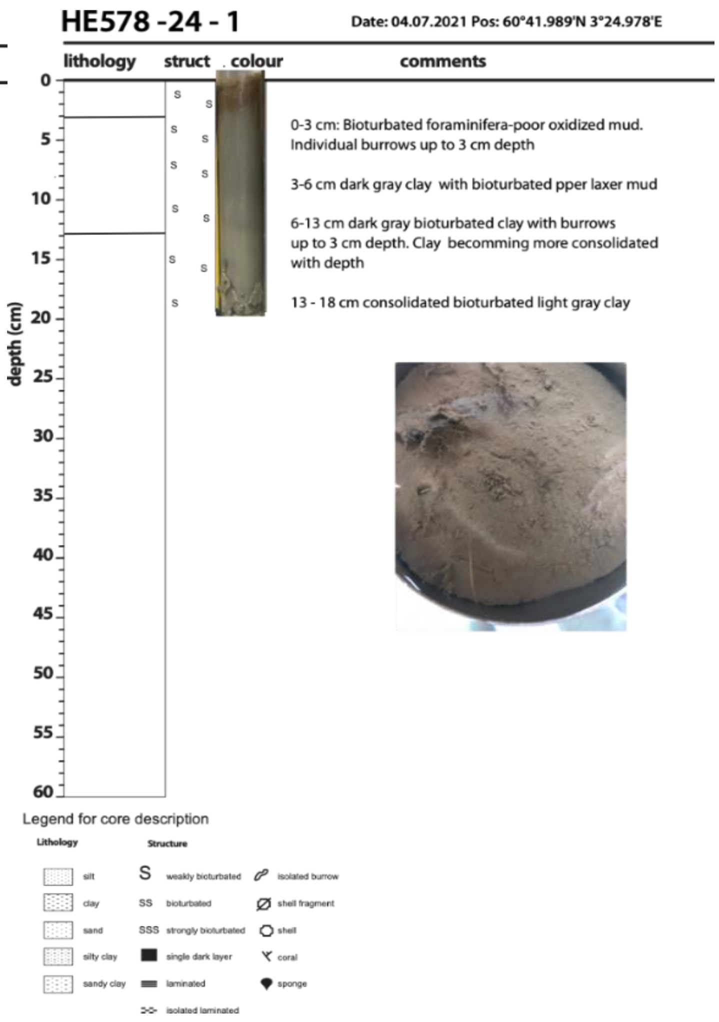
HE578 -24 - 1
Date: 04.07.2021 Pos: 60°41.989'N 3°24.978'E
lithology
struct
colour
comments
depth (cm)
0-3 cm: Bioturbated foraminifera-poor oxidized mud. Individual burrows up to 3 cm depth
3-6 cm dark gray clay with bioturbated pper laxer mud
6-13 cm dark gray bioturbated clay with burrows up to 3 cm depth. Clay becomming more consolidated with depth
13 - 18 cm consolidated bioturbated light gray clay
Legend for core description
Lithology
Structure
silt
clay
sand
silty clay
sandy clay
weakly bioturbated
bioturbated
strongly bioturbated
single dark layer
laminated
isolated laminated
isolated burrow
shell fragment
shell
coral
sponge

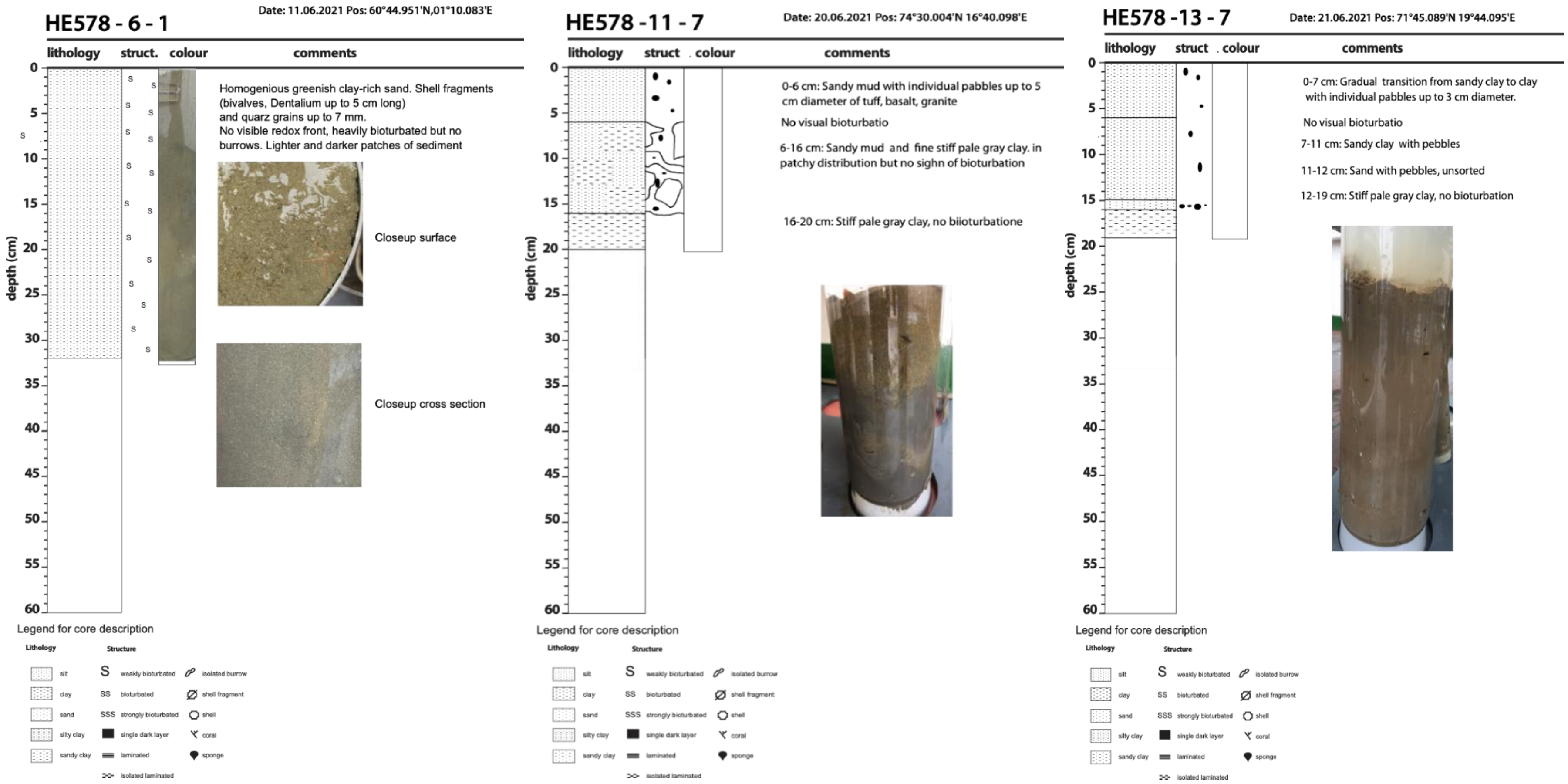

Figure S2. Lithology observations of the sediment cores.

Paragraph S1. Lithology description of the sediment cores.

Sediments collected along the Fedje-Shetland transect (Stations S1, S24, and S6) consisted of heavy bioturbated mud and clay-rich sand. At the Bjørnoya transect, sediments (S11) consisted of stiff dark grey clays covered with a transition zone of dark-grey clay and yellow sandy clay covered with a layer of yellow sandy clay with individual pebbles of different sizes that were rounded but not sorted. The sediments showed no signs of bioturbation. Examination of the palynological content of the sediments revealed that this clay was of terrestrial origin (containing triplets and monolete spores with no signs of marine palynomorphs). Overlaying sandy clays had a marine origin containing microfossils organic-walled dinoflagellate cysts, and benthic- and planktic foraminifera. At the Fugloya transect, sediments (S13) showed a gradual transition from grey clays to yellow sandy clays containing randomly distributed pebbles. In consisted of the sediments of the Bjørnoya transect, the individual pebbles of different sizes were rounded but not sorted.

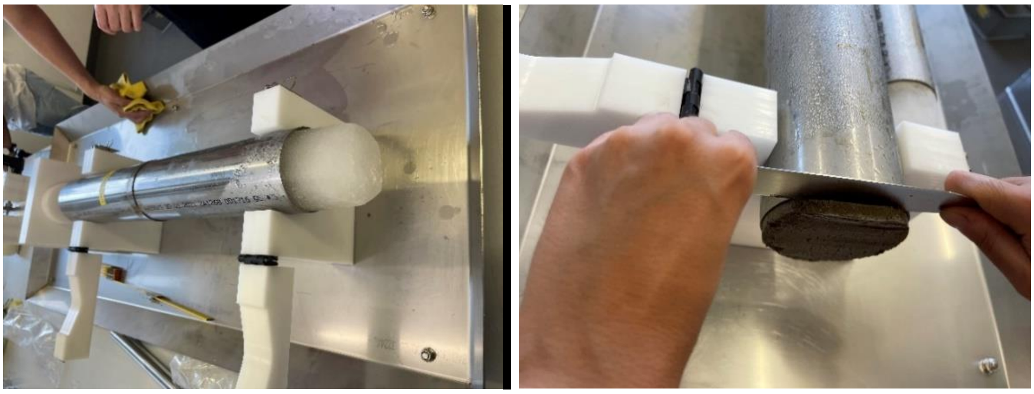

Figure S3. Slicing Sediment Core 4 of each station while frozen in the lab at MURUM, University of Bremen, Germany for total organic carbon and radiometric dating analysis.

Paragraph S2. Radiometric dating.

All samples were stored for a minimum of 28 days for equilibrium of the Radium 226 with his daughters ^{222}Rn, ^{214}Pb, and ^{214}Bi. Samples were measured by high-purity low-level germanium detector (BE 3830P-7500SL-ULB, Mirion Technologies (Canberra), Ruesselsheim, Germany) for the energy lines 46.54 keV (^{210}Pb), 661.66 keV (^{137}Cs), 295.21 keV, 351.92 keV (^{214}Pb), and 609.32 keV (^{214}Bi). Signal processing was done using a DSA LX (Mirion Technologies (Canberra), Ruesselsheim, Germany) together with the gamma spectrometry software InterWinner 8.0 (ITECH Instruments, Rognac, France). Background subtraction was done by measurements without any sample. Measurement time varied between 90 000 s – 600 000 s depending on the sample activity. For calibration, an artificial reference material was prepared with silica gel and reference solutions of ^{137}Cs and ^{226}Ra (Eckert & Ziegler Nuclitec GmbH, Braunschweig, Germany). With the determination of the daughters of ^{226}Ra, the supported

^{210}Pb (by ^{226}Ra decay) was provided. The unsupported ^{210}Pb, which represents the atmospheric fallout, was calculated by subtracting the supported ^{210}Pb from the total ^{210}Pb activity.

Paragraph S3. description of age calculation.

The age model of the sediment cores is based on the tephrochronology complemented by $^{210}Pb/^{137}Cs$ dating. The age of the sediments was calculated with a correction for the increasing compaction with depth in the upper sediments. At constant sedimentation rates over time, sediments in the upper part of the core contain more water compared to deeper sediment layers, resulting in a lower DBD. For correction, the relationship between core depth and DBD was calculated using the program Past V4.02, assuming an exponential model.

Based on this relationship, modelled DBD values have been calculated according to the following equations:

Core S1: $y = 11.046x$^$0.014092 – 10.756$

Core S24: $y = 44.518x$^$0.002377 – 44.012$

where y= modelled DBD, x = core depth (mm)

Based on this modelled DBD value, a modelled depth was calculated for the ^{210}Pb measurement points with the following equations:

Core S1: $y = 0.30319e(5.568x) – 0.79021$

Core S24: $y = 1.6478e(2.7447x) – 4.1912$

where y = compaction corrected depth (mm) and x = modelled DBD (g $cm^{–3}$)

Sedimentation rates were determined based on the compaction-adapted core depths using the ^{210}Pb data excluding the uppermost points (assuming a bioturbation depth of 3 cm). This was done using an exponential model with the program Past V4.02 using a ^{210}Pb half-life time of 22.2 years with the following equations:

Core S1: $y = 4568.7e(-0.84939x) + 20.08$

Core S24: $y = 4888e(-0.50962x) – 27.193$

where y = ^{210}Pb value and x = compaction adapted core depth (mm).

This results in a sedimentation rate of 26.79 compaction corrected cm $year^{–1}$ for core S1 and 16.56 compaction corrected cm $year^{–1}$ for core S24.

The age of the core was then determined based on the age per cm compacted and depth adapted for compaction with the following equations:

Core S1: $y = 2021– 26.79(x – 3.188343)$

Core S24: $y = 2021 – 16.56(x – 4.904117)$

where y = the estimated age of the sediment layer, x = depth adapted for compaction

Paragraph S4. Quality assurance and quality control.

Several measures were implemented to minimize the potential contamination of samples with MP particles. Unless otherwise specified, all laboratory containers used were glass or stainless steel and were thoroughly air-blown (Airbrush compressor AF186, Wildanger Technik GmbH, Germany) and rinsed with Milli-Q water before use. All polymeric items that could not be replaced with glass alternatives such as bottle caps and tubes were made of polytetrafluoroethylene (PTFE). All glass jars used for sampling were pre-cleaned in a laboratory glassware washer (Miele Professional PG 8583 CD, Germany) and rinsed with Milli-Q water and then packed. Materials such as sampling clothes, buckets, and cleaning tissue were used as reference materials for comparison (Figure S5). A dust box (DB1000, G4 prefiltration, HEPA-H14 final filtration, Q¼ 950 m^3 h^{-1}, Mocklinghoff Lufttechnik, Germany) was installed in each laboratory to remove airborne particles. All sample processing steps were performed on a laminar flow bench (ScanLaf Fortuna, 1800; LaboGene, Lillerød, Denmark), except for the addition of 97% H_2SO_4 and Tween 20, which was performed in a fume hood for safety reasons. The 10 µm stainless steel filter meshes (Ø 47 mm) used for sample processing were rinsed with Milli-Q water, soaked, and sonicated for 3 minutes (Bandelin Sonorex, Bandelin 250 electronic GmbH & Co.KG, Germany) before a final rinse with Milli-Q water before use. All solutions were pre-filtered using glass microfiber filters (GF/F, Ø 47 mm, 0.7 µm pore size, VWR, Germany), except for 97% H_2SO_4. Cotton lab coats were worn to minimize contamination from synthetic textiles, while nitrile gloves were worn during Fenton’s reaction for workplace safety. Procedural blanks (n=10 for sediment core S1, S24, S6, and S13; n=3 for sediment core S11; n=3 for overlying water) were processed and analysed concurrently with environmental samples. Additionally, acrylamide was excluded due to poor hit-quality matches of spectra.

Paragraph S5. Full details of the workflow of extraction of microplastics from sediment samples.

(1) First density separation (Overflow). The rehydrated sample was transferred into a glass straight-neck bottle (300 ml, Duran DWK Life Sciences GmbH, Wertheim/Main, Germany). The bottle was then filled to 4–5 cm below the rim with pre-filtered NaBr and capped with an aluminium cap. To disaggregate sediment agglomerates, the bottle was then submerged in an ultrasonic bath (BactoSonic, Bandelin electronic GmbH & Co.KG) and sonicated at 80 W/40 kHz for 15 min. The samples were then stirred with a cage stirrer on top of a magnetic stirrer

(C-MAG HS 7 control; IKA-Werke GmbH & Co. KG, Staufen, Germany) for 1 h at 400 rpm. Before placing the bottles in the laminar flow cabinet (ScanLaf Fortuna, 1800; LaboGene, Lillerød, Denmark), the external walls of the bottles were cleaned using an airbrush (Airbrushgun Typ 180, WilTec Wildanger Technik GmbH, Germany), to remove potential airborne contamination and rinsed with Milli-Q water. The bottles were then placed into PTFE evaporating basins with flat bottoms (height 36 mm, Ø ext. 132 mm, VWR International GmbH, Germany) and filled to 3 cm below the rim with pre-filtered NaBr, then the sample was left settled to allow the density separation process until the liquid column was clear. The settling time was between 4 – 6 h. To separate lighter materials from the sediment, 90 mL of NaBr solution was carefully added to each sample by using glass syringes (50 mL, Carl Roth GmbH+Co. KG, Germany), positioned 5 cm below the bottle brim to avoid sediment resuspension. This allowed the overflow of the lighter materials, which were subsequently collected in the basin. Following the overflow, the excess solution was partially removed from the bottle to prevent it from ending in the evaporation basins [1]. Subsequently, the external walls and the bottom of the bottles were then rinsed carefully with milli-Q water and the rinsing water was collected in the evaporating basins. Afterwards, all collected materials in the basin were then concentrated on a stainless-steel filter (10 µm) and flushed with ~ 500 ml Milli-Q water to remove any NaBr residue.

(2) **Fenton's treatment.** Fenton's treatment was applied to remove organic material present in the sample and followed the procedures outlined in Al-Azzawi et al. 2020[2] with minor modifications. Briefly, 15 mL of $FeSO_4$ (20 g L^{-1}) was first added to the beaker placed in an ice bath, followed by the slow addition of 70 mL of H_2O_2 (30%, Fa. Bernd Kraft GmbH, Germany) over 10 min with the help of a dosing pump (flow rate: 7 mL min^{-1}, SIMDOS 02 FEM 1.02 S, KNF, Germany). After a further 10 min, 4 mL of H_2SO_4 (97%) was slowly added dropwise to the sample to remove precipitated iron formed during the reaction and to clear the sample. 10 mL of Tween 20 (0.1%, VWR International, France) was then added to dilute the sample and prevent particles from adhering to the glass walls. During the Fenton reaction, a cage stirrer was placed on top of the filter and the ice bath was placed on a stir plate (C-MAG HS 7 control, IKA, Germany), the temperature was monitored by a thermometer connected to the stir plate and controlled below 40°C by the addition of ice.[3] Afterwards, the treated sample was vacuum filtered onto a new 10 µm stainless steel filter and flushed with ~ 500 mL Milli-Q water to remove any Fenton residue.

(3) Second density separation. After the Fenton reaction, to remove potential heavy inorganic material, a second density separation was applied. The treated samples were removed from the filters by placing them in 150 mL beakers containing ~40 mL of NaBr solution and sonicated

for 5 min at 80 W/40 kHz (BactoSonic, Bandelin electronic GmbH & Co.KG). Afterwards, the filters and the beaker were rinsed with NaBr solution and transferred to 50 mL separation funnels and placed under a laminar flow cabinet. Before allowing the samples to settle, the separation funnels were shaken carefully by hand for 30 sec. After 12 h (overnight), the lower fraction (~15 mL) in the funnel was discarded. New NaBr were added to reach the initial volume. This procedure was repeated twice after a further 4 h and 4 h settling time. Subsequently, the remaining sample solutions were filtered onto new stainless-steel filters (10 µm) and flushed with ~500 mL Milli-Q water to remove any NaBr residue. Afterwards, the sample material from the filters was retained in a glass neck bottle (100 mL) and stored at 4°C until further analysis.

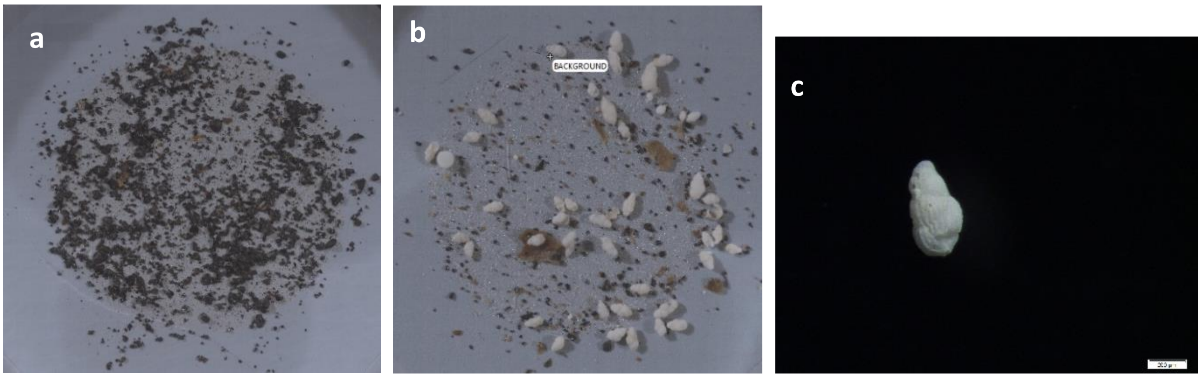

Figure S4. **a** refers to coal particles on one of the Anodisc filters from S11. **b** refers to shell residues on one of the Anodisc filters. **c** refers to one shell picked from the Anodisc and checked under the stereomicroscope.

Paragraph S6. Microplastic identification.

Putative MPs concentrated on the Anodisc filters were measured by a μFTIR-microscope (Hyperion 3000) connected to a Tensor 27 spectrometer (Bruker Optik GmbH, Germany) equipped with a 3.5× objective and a 64 × 64 focal plane array (FPA) detector with a pixel size of 11 μm, which sets the lower detection limit of the present analysis. A spectral range of 1250–3600 cm^{-1} with 32 co-added scans collected at a resolution of 8 cm^{-1} was used. [1, 4] A grid of 20–26 measurement fields was applied to cover all particles in the filtration area. Due to the presence of coal particles and shell residues in certain (Figure S4), a BaF_2 window was not used to cover the Anodisc filters. Consequently, the MP particles were not morphologically categorized into elongated particles (fibre-like MPs with an aspect ratio of 3:1 or higher [5]) and particle-like MPs. The IR spectra obtained were processed with OPUS 8.8 software. Subsequently, automatic identification and quantification of MPs were conducted using an updated version of siMPle,[6, 7] utilising a reference database originally designed by Primpke, et al. [8] and updated by Roscher, et al. [9] The final tabular data, including the size of each MP particle and the specified polymer clusters, were obtained directly from the siMPle spectra analysis (version 1.3.2.1, available upon request). The image analysis through MPAPP was performed based on the spectral matching process of Primpke et al., [7] using matching scores from a minimum of 600 to a maximum of 2000. The database is grouped into polymer types for material identification with individual minimum matching scores. In this study, the polymer types proposed by Lorenz et al., [10] and Roscher et al., [9] were applied for the reliable identification of the spectra to achieve comparable results to previous studies.

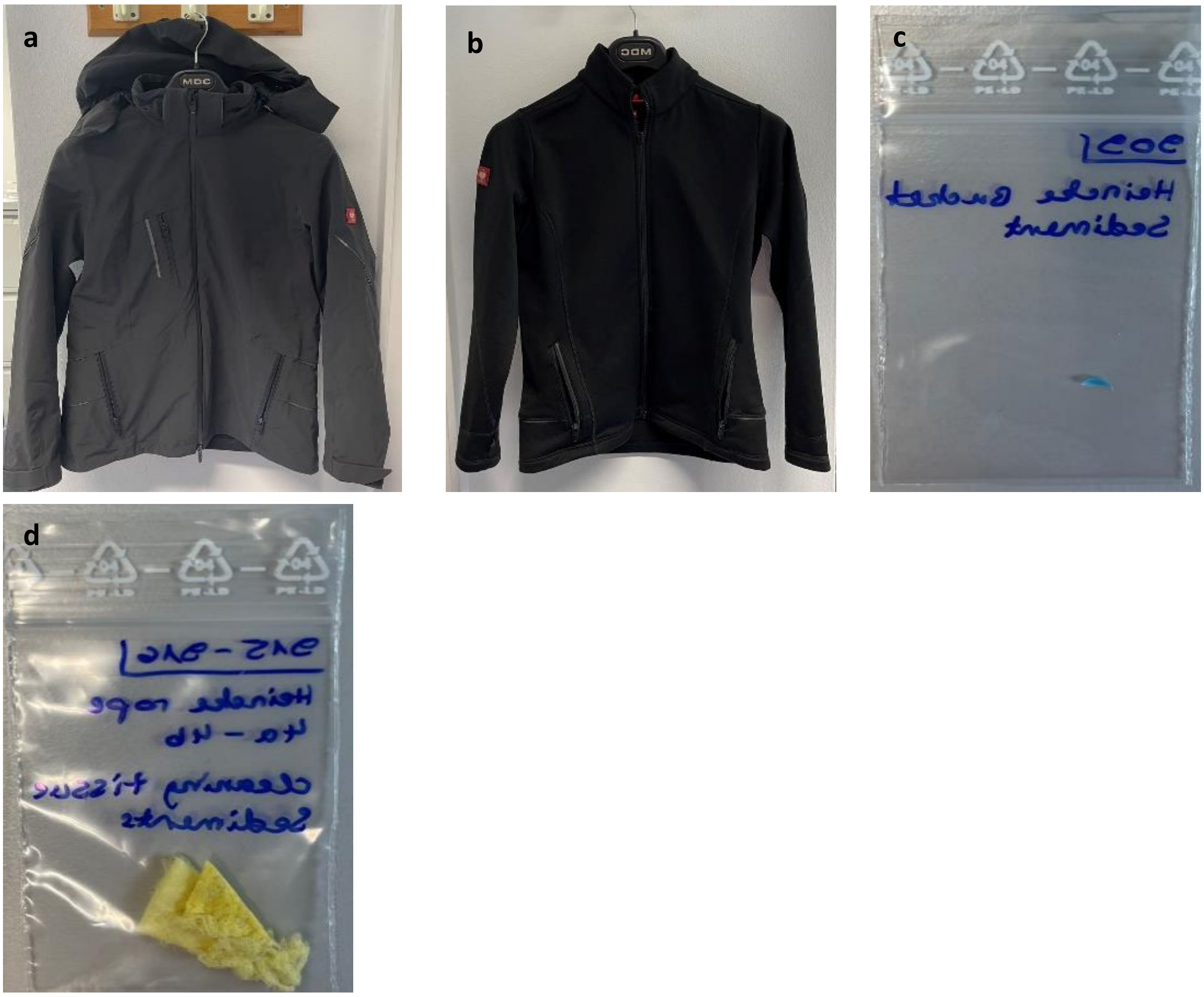

Figure S5. Clothes, bucket, and cleaning tissue used during sediment sampling. a refers to outside material of the sampling jacket, polyamide. **b** refers to inside material of the sampling jacket, polyester. **c** refers to bucket used for cleaning cutting knife, polypropylene. **d** refers to cleaning tissue for cleaning knife, polypropylene.

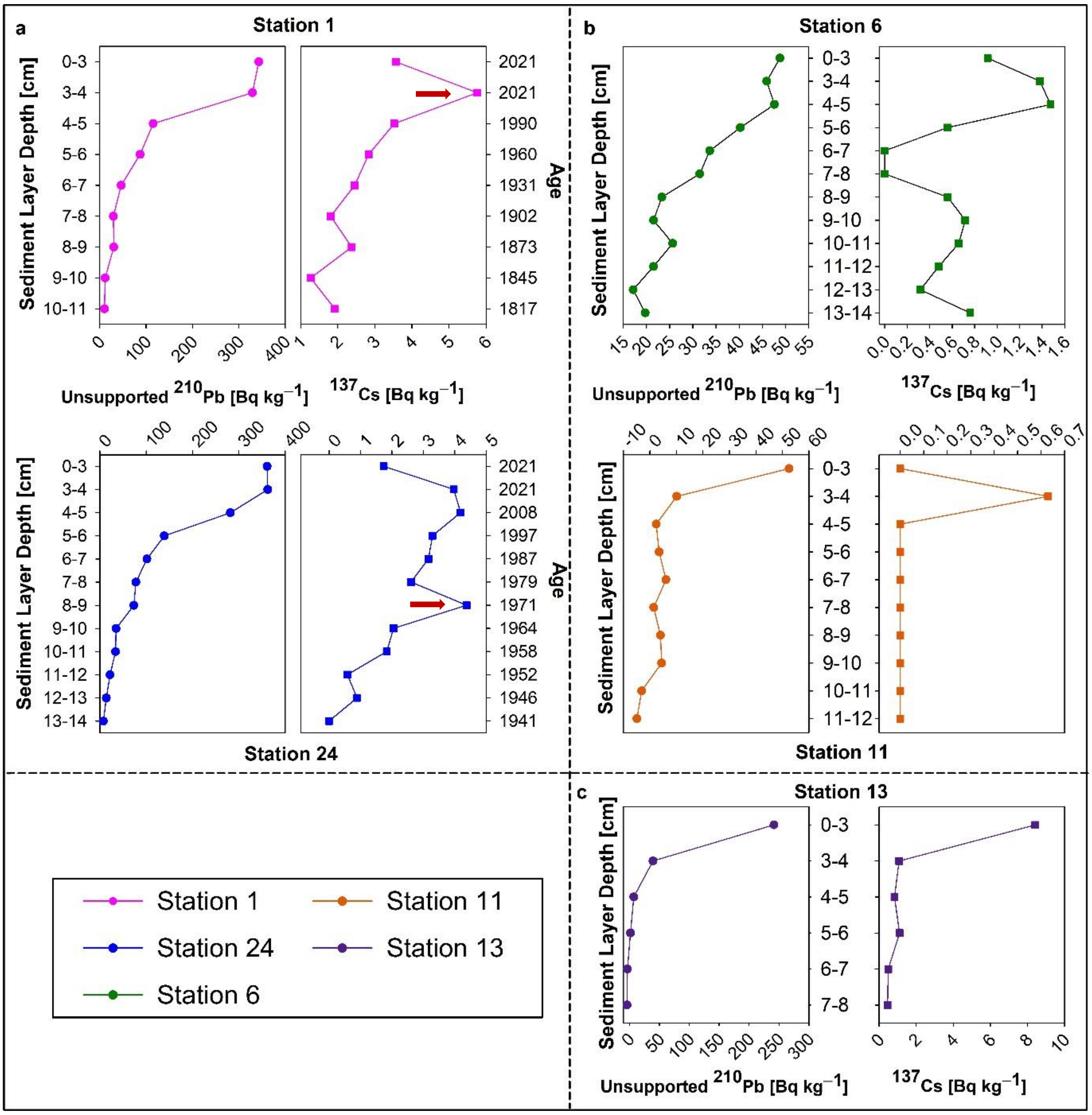

Figure S6. Vertical profile of unsupported ^{210}Pb and ^{137}Cs of a) the two cores (S1 and S24) whose ages were estimated, **b)** the two cores whose ages were not calculated due to potential disturbance from fishing activities**, c)** the station with low sedimentation rate.

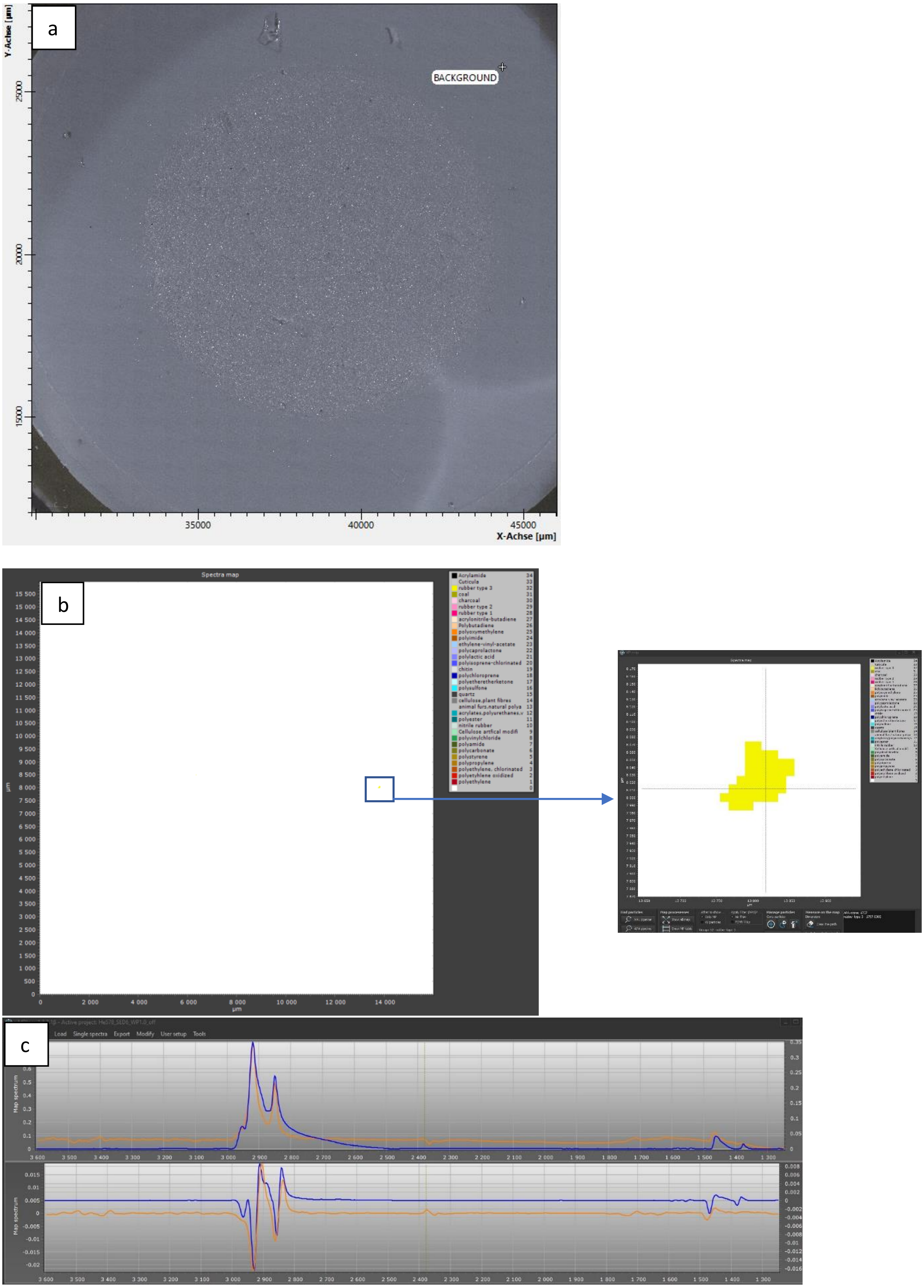

Figure S7. a refers to the visual image of the Anodisc filter**, b** refers to image analysis in siMPle, and **c** refers to the spectra of the rubber type 3 identified in the water-sediment interphase at station 6.

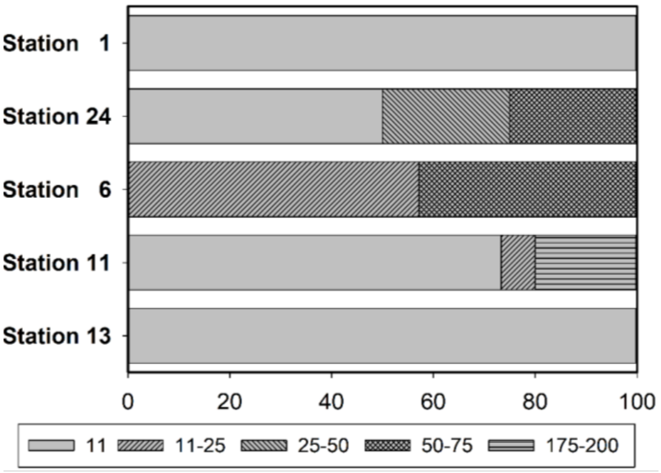

Figure S8. Percentage of each size class [µm] in overlying waters of different sediment cores

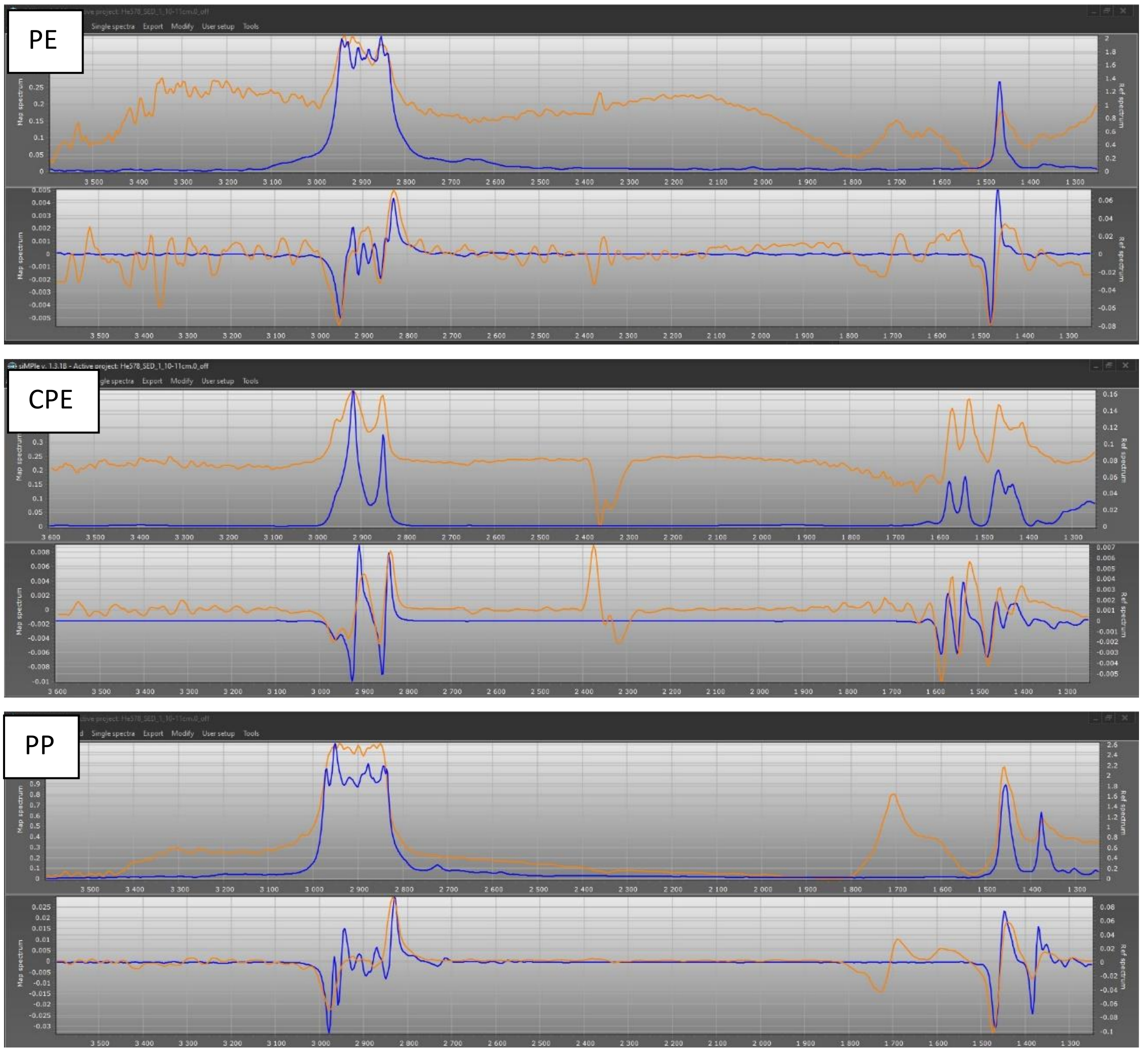

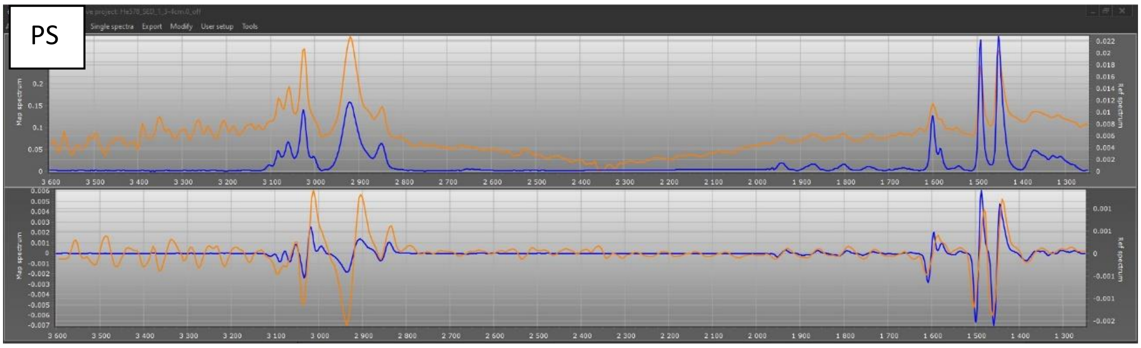
PS

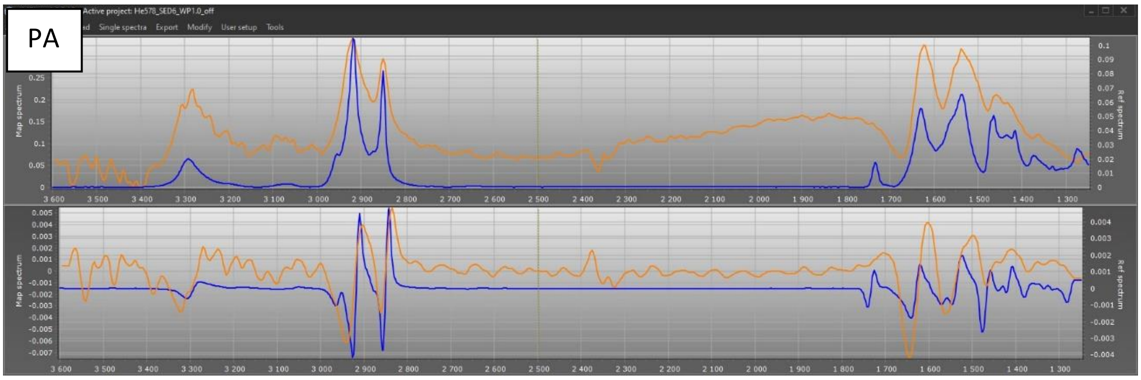
PA

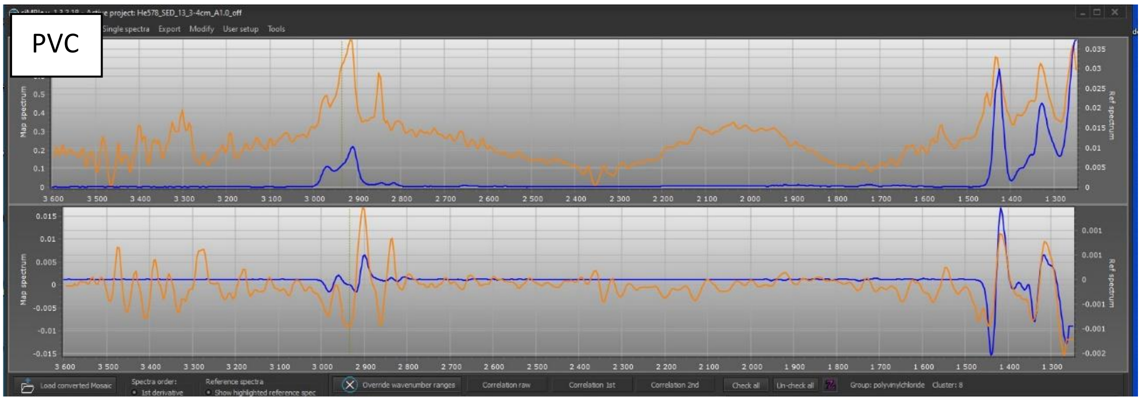
PVC

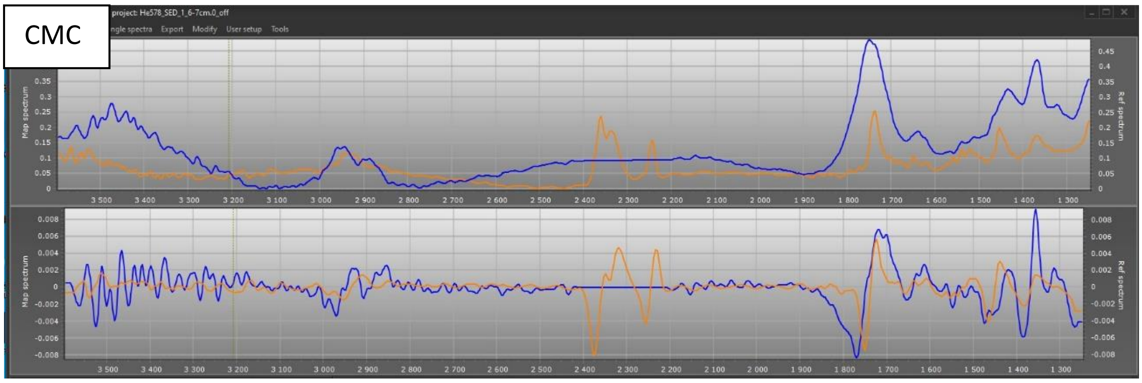
CMC

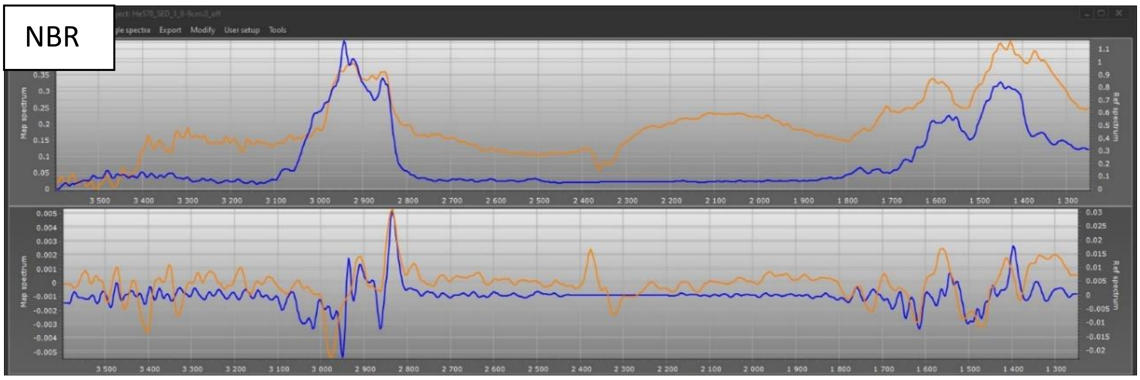
NBR

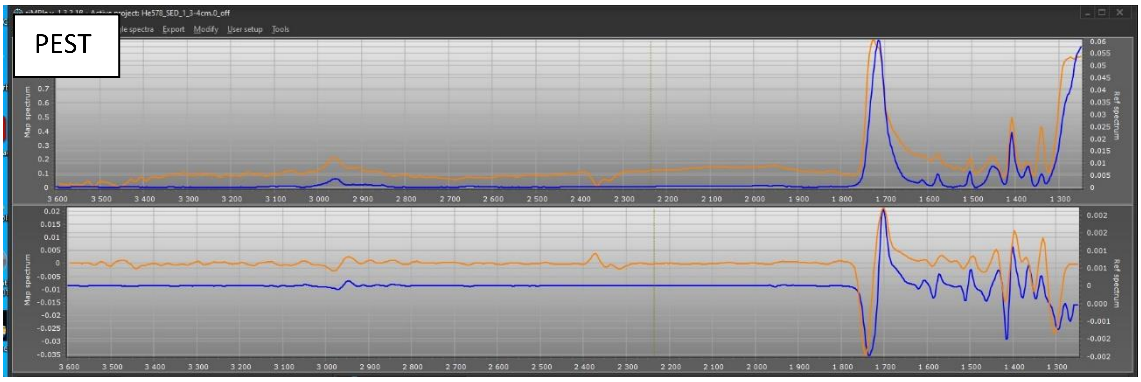
PEST

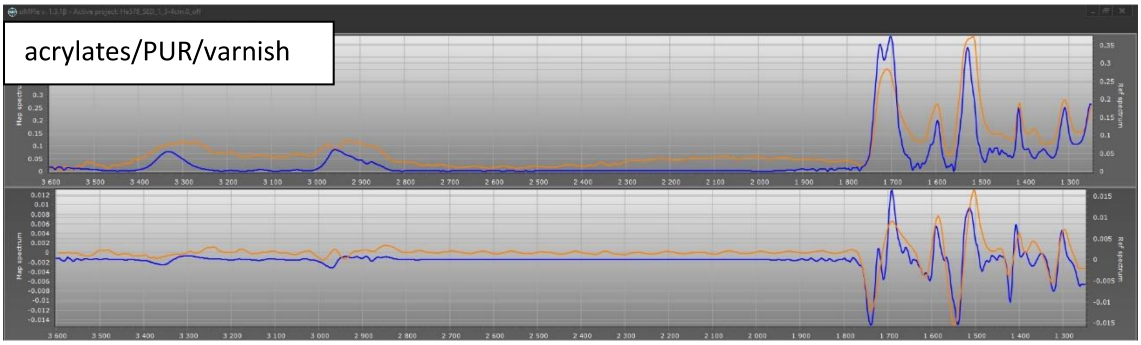
acrylates/PUR/varnish

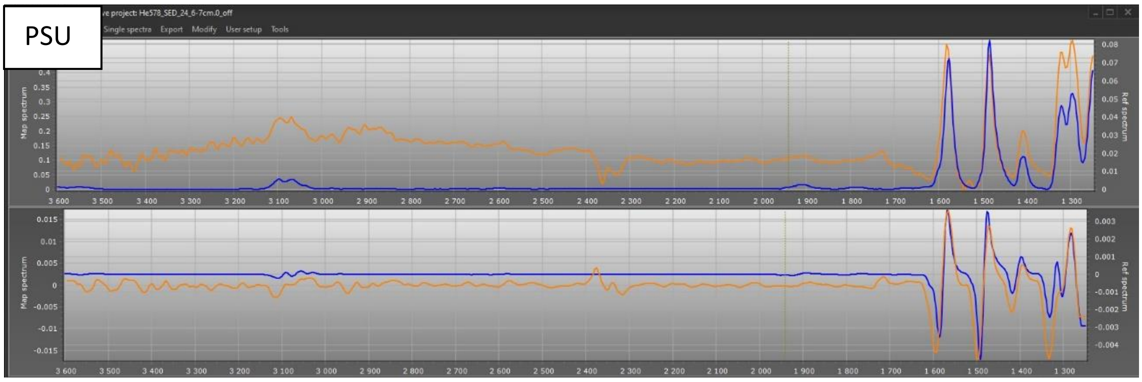
PSU

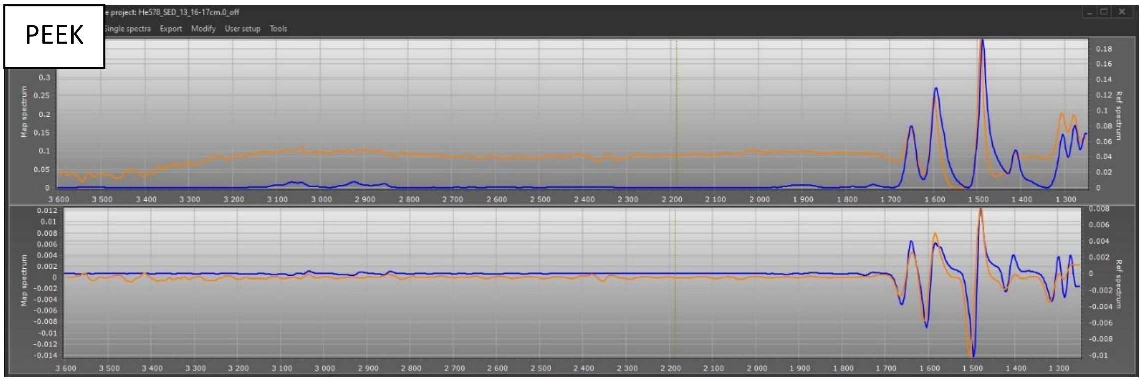
PEEK
project: He578_SED_13_16-17cm.0_off

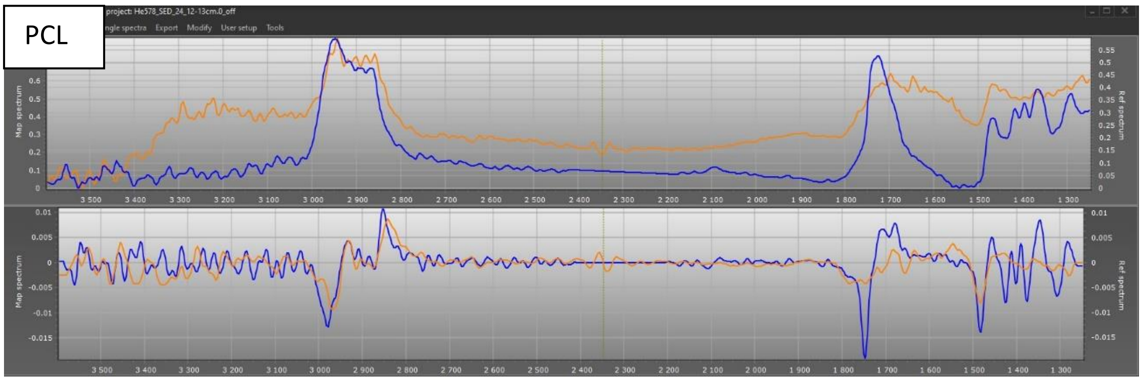
PCL
project: He578_SED_24_12-13cm.0_off

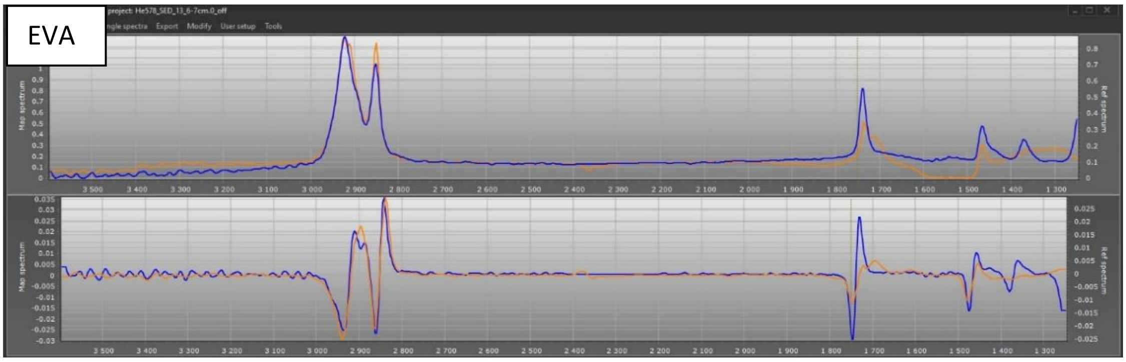
EVA
project: He578_SED_13_6-7cm.0_off

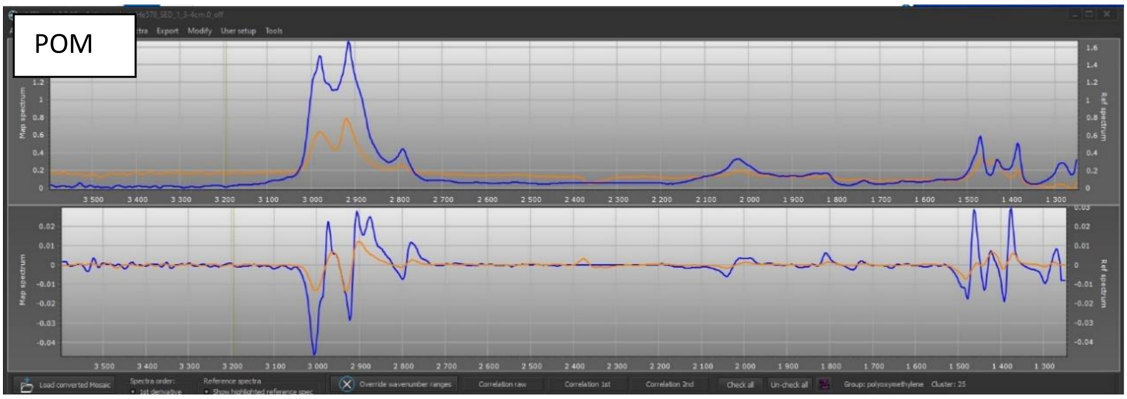
POM

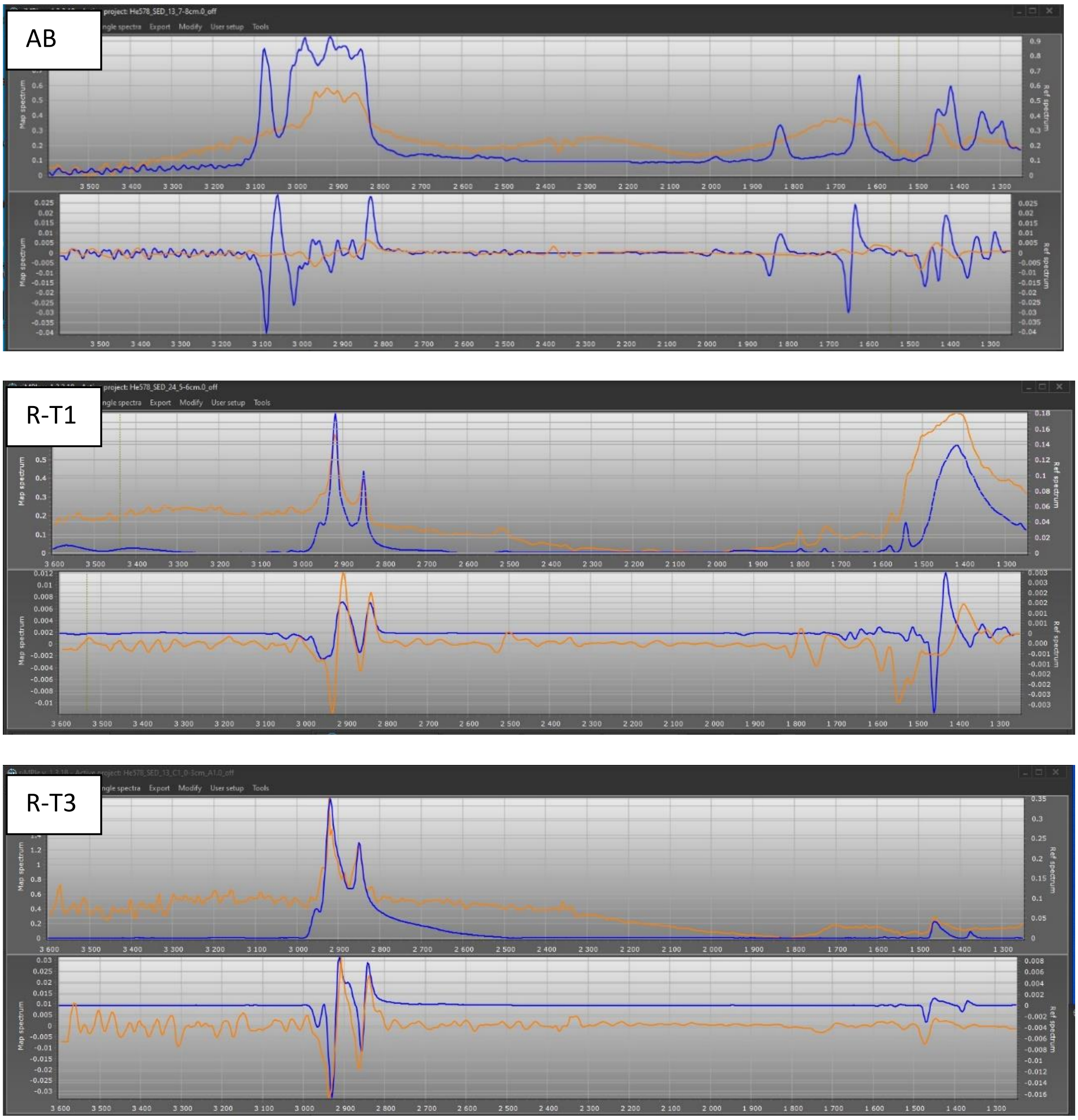

Figure S9. Spectra of the 18 polymer types detected in all sediment cores. The software graphical user interface with a loaded reference (blue) and a sample spectrum (orange) using the match single spectra function of siMPle, more details refer to Primpke et al., [6]. PE: polyethylene, CPE: chlorinated polyethylene, PP: polypropylene, PS: polystyrene, PA: polyamide, PVC: polyvinyl chloride, CMC: chemically modified cellulose, NBR: nitrile rubber, PEST: polyester, PUR: polyurethane, PSU: polysulfone, PEEK: polyether ether ketone, PCL: polycaprolactone, EVA: ethylene vinyl acetate, POM: polyoxymethylene, AB: acrylonitrile butadiene, R-T1: rubber type 1. R-T3: rubber type 3.

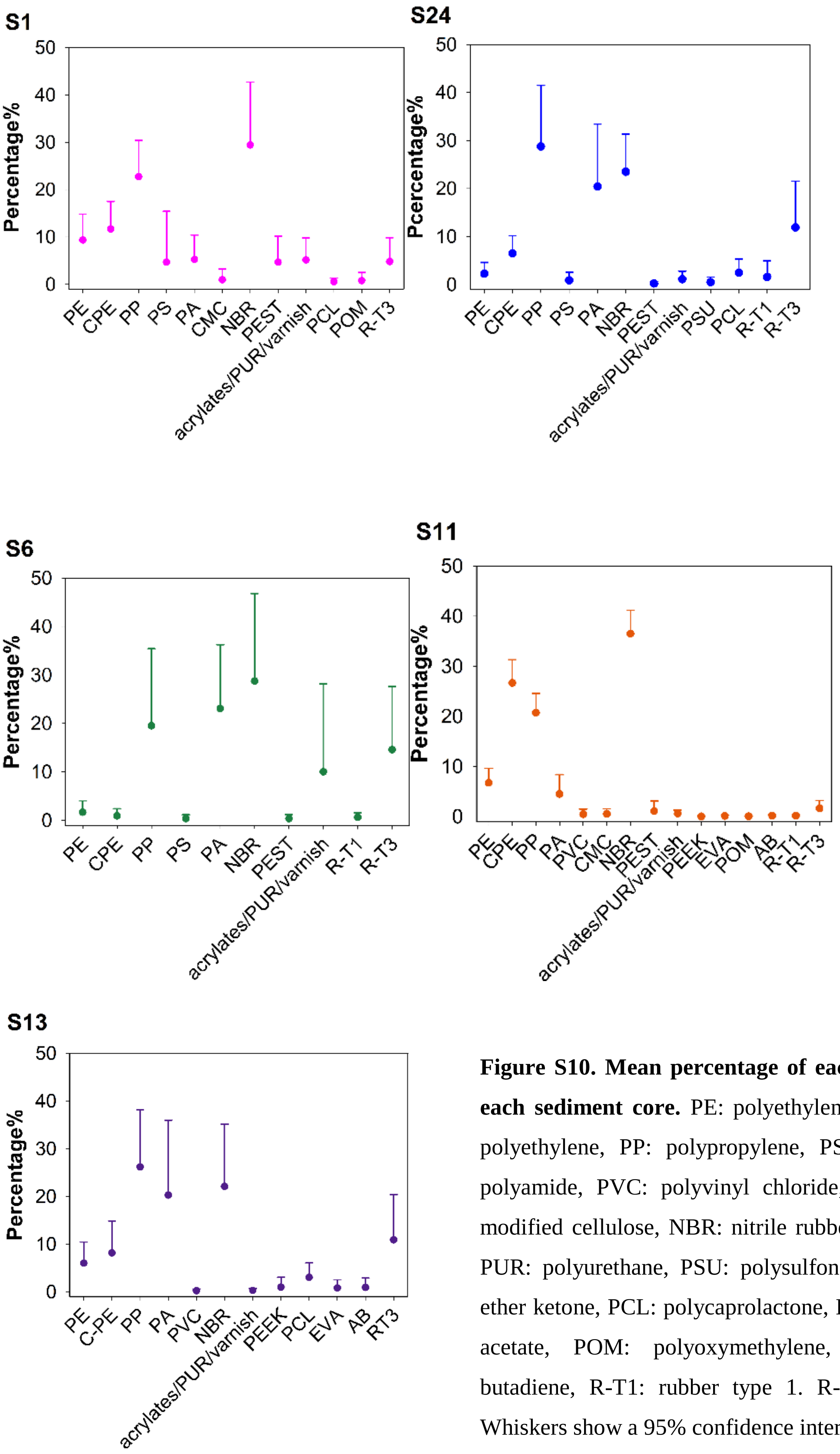

Figure S10. Mean percentage of each polymer type in each sediment core. PE: polyethylene, CPE: chlorinated polyethylene, PP: polypropylene, PS: polystyrene, PA: polyamide, PVC: polyvinyl chloride, CMC: chemically modified cellulose, NBR: nitrile rubber, PEST: polyester, PUR: polyurethane, PSU: polysulfone, PEEK: polyether ether ketone, PCL: polycaprolactone, EVA: ethylene vinyl acetate, POM: polyoxymethylene, AB: acrylonitrile butadiene, R-T1: rubber type 1. R-T3: rubber type 3. Whiskers show a 95% confidence interval.

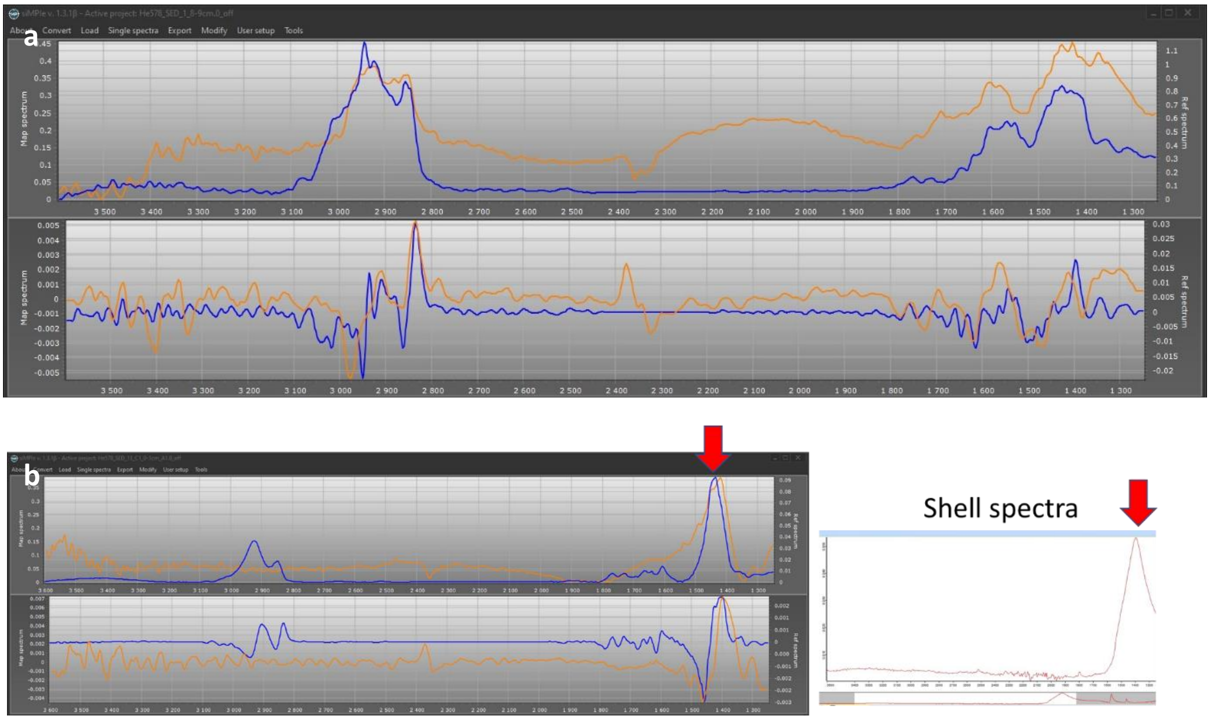

Figure S11. a refers to good nitrile rubber (NBR) spectra detected in sediment samples. **b** refers to an inadequate nitrile rubber spectra, which was a particle assigned to the polymer "nitrile rubber" but appears to have a similar peak as shell residue.

Table S1. Please see the separate metadata Excel sheet for Sampling points, each polymer concentration, MP concentration per layer, sampling depths, numbers of Anosidc filters per sample, sediment dry weight used for analysis, TOC%, wet density, porosity, dry bulk density, unsupported ^{210}Pb and ^{137}Cs activities, estimated age of core S1 and S24 and polymer diversity

Table S2. Averaged procedural blanks in overlying water and sediment samples (Numbers of polymers).

	PE	PA	PVC	PEST	CMC	acrylates/PUR/varnish	R-T3
Overlying Water	-	1.0	-	-	-	0.3	-
Cores S1, S24, S6, and S13	0.1	0.3	0.1	-	0.1	0.4	0.8
Core S11	-	1	-	0.33	-	-	0.33

Polyethylene (PE), Polyamide (PA), Polyvinyl chloride (PVC), Polyester (PEST), Chemically modified cellulose (CMC), polyurethanes (PUR), Rubbertype 3 (R-T3)

Table S3 Spearman's Rank Correlation of MP concentrations, smallest size class percentage, polymer diversity and ancillary data (depth, total organic carbon, wet density, porosity and dry bulk density) in post-1950s layers in station 1.

**Station 1_post 1950s**								
Variable	Spearman Rank Order Correlations (He578_SED_core S1_1950s.sta)<br>MD pairwise deleted<br>Marked correlations are significant at *p* <.05000							
	depth	MP concentration	smallest size class percentage	polymer diversity	TOC	Wet density	porosity	dry bulk density
depth	1.000000	-0.800000	0.000000	0.210819	0.000000	0.400000	-0.800000	1.000000
MP concentration	-0.800000	1.000000	-0.400000	0.316228	-0.400000	0.200000	0.600000	-0.800000
smallest size class percentage	0.000000	-0.400000	1.000000	-0.948683	1.000000	-0.800000	-0.400000	0.000000
polymer diversity	0.210819	0.316228	-0.948683	1.000000	-0.948683	0.948683	0.105409	0.210819
TOC	0.000000	-0.400000	1.000000	-0.948683	1.000000	-0.800000	-0.400000	0.000000
Wet density	0.400000	0.200000	-0.800000	0.948683	-0.800000	1.000000	-0.200000	0.400000
porosity	-0.800000	0.600000	-0.400000	0.105409	-0.400000	-0.200000	1.000000	-0.800000
dry bulk density	1.000000	-0.800000	0.000000	0.210819	0.000000	0.400000	-0.800000	1.000000

Table S4 Spearman's Rank Correlation of MP concentrations, smallest size class percentage, polymer diversity and ancillary data (depth, total organic carbon, wet density, porosity and dry bulk density) in all sediment cores.

Station 1 (all layers)								
	Spearman Rank Order Correlations (He578_SED_core S1.sta) MD pairwise deleted Marked correlations are significant at *p* <.05000							
Variable	Depth	MP concentration	smallest size class percentage	diversity	TOC	wet density	porosity	dry bulk density
Depth	1.000000	-0.800000	0.200000	-0.594573	-0.663889	0.833333	-0.583333	0.900000
MP concentration	**-0.800000**	1.000000	-0.400000	0.865628	0.310935	-0.583333	0.516667	-0.683333
smallest size class percentage	0.200000	-0.400000	1.000000	-0.655779	0.310935	-0.266667	-0.716667	0.000000
diversity	-0.594573	**0.865628**	-0.655779	1.000000	0.132263	-0.332261	0.515879	-0.463417
TOC	-0.663889	0.310935	0.310935	0.132263	1.000000	-0.731118	0.252110	-0.571449
wet density	0.833333	-0.583333	-0.266667	-0.332261	-0.731118	1.000000	-0.100000	0.933333
porosity	-0.583333	0.516667	**-0.716667**	0.515879	0.252110	-0.100000	1.000000	-0.250000
dry bulk density	0.900000	**-0.683333**	0.000000	-0.463417	-0.571449	0.933333	-0.250000	1.00000

Variable	**Station 24** Spearman Rank Order Correlations (He578_SED_core S24.sta)<br>MD pairwise deleted<br>Marked correlations are significant at *p* <.05000							
	Depth	MP concentration	smallest size class percentage	polymer diversity	TOC	wet density	porosity	dry bulk density
Depth	1.000000	-0.629371	0.111888	-0.751009	-0.979021	-0.209790	-0.776224	0.636364
MP concentration	**-0.629371**	1.000000	0.342657	0.619315	0.706294	0.125874	0.475524	-0.503497
smallest size class percentage	0.111888	0.342657	1.000000	-0.174405	-0.069930	-0.349650	-0.181818	-0.041958
polymer diversity	**-0.751009**	**0.619315**	-0.174405	1.000000	0.729653	0.242031	0.565926	-0.484062
TOC	-0.979021	**0.706294**	-0.069930	0.729653	1.000000	0.230769	0.769231	-0.643357
wet density	-0.209790	0.125874	-0.349650	0.242031	0.230769	1.000000	0.671329	0.503497
porosity	-0.776224	0.475524	-0.181818	0.565926	0.769231	0.671329	1.000000	-0.111888
dry bulk density	0.636364	-0.503497	-0.041958	-0.484062	-0.643357	0.503497	-0.111888	1.000000

Station 6								
	Spearman Rank Order Correlations (He578_SED_core S6.sta)<br>MD pairwise deleted<br>Marked correlations are significant at *p* <.05000							
Variable	Depth	MP concentration	smallest size class percentage	polymer diversity	TOC	Wet density	porosity	dry bulk density
depth	1.000000	-0.272727	0.328671	-0.320256	-0.860140	0.132867	-0.475524	0.363636
MP concentration	-0.272727	1.000000	-0.076923	0.578645	0.188811	-0.083916	0.258741	-0.195804
smallest size class percentage	0.328671	-0.076923	1.000000	-0.138292	-0.622378	-0.251748	-0.657343	-0.097902
polymer diveristy	-0.320256	**0.578645**	-0.138292	1.000000	0.141932	-0.090982	0.232914	-0.185603
TOC	-0.860140	0.188811	**-0.622378**	0.141932	1.000000	-0.055944	0.510490	-0.314685
Wet density	0.132867	-0.083916	-0.251748	-0.090982	-0.055944	1.000000	0.587413	0.958042
porosity	-0.475524	0.258741	-0.657343	0.232914	0.510490	0.587413	1.000000	0.405594
dry bulk density	0.363636	-0.195804	-0.097902	-0.185603	-0.314685	0.958042	0.405594	1.000000

**Station 11**								
Variable	Spearman Rank Order Correlations (He578_SED_core S11.sta)<br>MD pairwise deleted<br>Marked correlations are significant at *p* <.05000							
	depth	MP concentration	smallest size class percentage	Polymer diversity	TOC	Wet density	porosity	dry bulk density
depth	1.000000	0.654545	-0.836364	0.623240	0.963636	0.454545	0.260606	0.515152
MP concentration	**0.654545**	1.000000	-0.481818	0.370195	0.660606	0.369697	0.139394	0.563636
smallest size class percentage	**-0.836364**	-0.481818	1.000000	-0.604496	-0.830303	-0.369697	-0.200000	-0.393939
Polymer diversity	**0.623240**	0.370195	-0.604496	1.000000	0.462726	-0.075037	0.362677	-0.106302
TOC	**0.963636**	**0.660606**	**-0.830303**	0.462726	1.000000	0.381818	0.200000	0.442424
Wet density	0.454545	0.369697	-0.369697	-0.075037	0.381818	1.000000	0.684848	0.951515
porosity	0.260606	0.139394	-0.200000	0.362677	0.200000	0.684848	1.000000	0.515152
dry bulk density	0.515152	0.563636	-0.393939	-0.106302	0.442424	0.951515	0.515152	1.000000

Station 13								
Variable	Spearman Rank Order Correlations (HE578_SED_coreS13.sta)<br>MD pairwise deleted<br>Marked correlations are significant at *p* <.05000							
	depth	MP concentration	smallest size class percentage	polymer diversity	TOC	Wet density	porosity	dry bulk density
depth	1.000000	-0.220588	0.073529	-0.449274	-0.977941	0.696078	-0.867647	0.889706
MP concentration	-0.220588	1.000000	0.213235	0.674551	0.151961	-0.171569	0.198529	-0.220588
smallest size class percentage	0.073529	0.213235	1.000000	0.072959	-0.073529	0.245098	0.102941	0.026961
polymer diversity	-0.449274	**0.674551**	0.072959	1.000000	0.427514	-0.298236	0.357115	-0.412154
TOC	-0.977941	0.151961	-0.073529	0.427514	1.000000	-0.732843	0.838235	-0.906863
Wet density	0.696078	-0.171569	0.245098	-0.298236	-0.732843	1.000000	-0.367647	0.906863
porosity	-0.867647	0.198529	0.102941	0.357115	0.838235	-0.367647	1.000000	-0.674020
dry bulk density	0.889706	-0.220588	0.026961	-0.412154	-0.906863	0.906863	-0.674020	1.000000

References

1. Abel, S. M.; Primpke, S.; Wu, F.; Brandt, A.; Gerdts, G., Human footprints at hadal depths: interlayer and intralayer comparison of sediment cores from the Kuril Kamchatka trench. *Sci. Total. Environ.* **2022,** *838*, (Pt 2), 156035.
2. Al-Azzawi, M. S. M.; Kefer, S.; Weißer, J.; Reichel, J.; Schwaller, C.; Glas, K.; Knoop, O.; Drewes, J. E., Validation of Sample Preparation Methods for Microplastic Analysis in Wastewater Matrices—Reproducibility and Standardization. *Water* **2020,** *12*, (9), 2445.
3. Wu, F.; Reding, L.; Starkenburg, M.; Leistenschneider, C.; Primpke, S.; Vianello, A.; Zonneveld, K. A. F.; Huserbraten, M. B. O.; Versteegh, G. J. M.; Gerdts, G., Spatial distribution of small microplastics in the Norwegian Coastal Current. *Sci. Total. Environ.* **2024,** *942*, 173808.
4. Roscher, L.; Fehres, A.; Reisel, L.; Halbach, M.; Scholz-Bottcher, B.; Gerriets, M.; Badewien, T. H.; Shiravani, G.; Wurpts, A.; Primpke, S.; Gerdts, G., Microplastic pollution in the Weser estuary and the German North Sea. *Environ. Pollut.* **2021,** *288*, 117681.
5. Primpke, S.; A. Dias, P.; Gerdts, G., Automated identification and quantification of microfibres and microplastics. *Anal. Methods.* **2019,** *11*, (16), 2138-2147.
6. Primpke, S.; Cross, R. K.; Mintenig, S. M.; Simon, M.; Vianello, A.; Gerdts, G.; Vollertsen, J., EXPRESS: Toward the Systematic Identification of Microplastics in the Environment: Evaluation of a New Independent Software Tool (siMPle) for Spectroscopic Analysis. *Appl. Spectrosc.* **2020**, 3702820917760.
7. Primpke, S.; Lorenz, C.; Rascher-Friesenhausen, R.; Gerdts, G., An automated approach for microplastics analysis using focal plane array (FPA) FTIR microscopy and image analysis. *Anal. Methods.* **2017,** *9*, (9), 1499-1511.
8. Primpke, S.; Wirth, M.; Lorenz, C.; Gerdts, G., Reference database design for the automated analysis of microplastic samples based on Fourier transform infrared (FTIR) spectroscopy. *Analytical and Bioanalytical Chemistry* **2018,** *410*, (21), 5131-5141.
9. Roscher, L.; Halbach, M.; Nguyen, M. T.; Hebeler, M.; Luschtinetz, F.; Scholz-Bottcher, B. M.; Primpke, S.; Gerdts, G., Microplastics in two German wastewater treatment plants: Year-long effluent analysis with FTIR and Py-GC/MS. *Sci. Total. Environ.* **2022,** *817*, 152619.
10. Lorenz, C.; Roscher, L.; Meyer, M. S.; Hildebrandt, L.; Prume, J.; Loder, M. G. J.; Primpke, S.; Gerdts, G., Spatial distribution of microplastics in sediments and surface waters of the southern North Sea. *Environ. Pollut.* **2019,** *252*, (Pt B), 1719-1729.
